# Time-restricted feeding is an intervention against excessive dark-phase sleepiness induced by obesogenic diet

**DOI:** 10.1093/nsr/nwac222

**Published:** 2022-10-16

**Authors:** Xu Wang, Keke Xing, Mengge He, Ting He, Xinkuan Xiang, Tao Chen, Luoying Zhang, Haohong Li

**Affiliations:** Britton Chance Center for Biomedical Photonics, Wuhan National Laboratory for Optoelectronics, Huazhong University of Science and Technology, Wuhan 430074, China; MOE Key Laboratory for Biomedical Photonics, Collaborative Innovation Center for Biomedical Engineering, School of Engineering Sciences, Huazhong University of Science and Technology, Wuhan 430074, China; Department of Anatomy, Histology & Embryology, Fourth Military Medical University, Xi’an 710032, China; Britton Chance Center for Biomedical Photonics, Wuhan National Laboratory for Optoelectronics, Huazhong University of Science and Technology, Wuhan 430074, China; MOE Key Laboratory for Biomedical Photonics, Collaborative Innovation Center for Biomedical Engineering, School of Engineering Sciences, Huazhong University of Science and Technology, Wuhan 430074, China; Britton Chance Center for Biomedical Photonics, Wuhan National Laboratory for Optoelectronics, Huazhong University of Science and Technology, Wuhan 430074, China; MOE Key Laboratory for Biomedical Photonics, Collaborative Innovation Center for Biomedical Engineering, School of Engineering Sciences, Huazhong University of Science and Technology, Wuhan 430074, China; Britton Chance Center for Biomedical Photonics, Wuhan National Laboratory for Optoelectronics, Huazhong University of Science and Technology, Wuhan 430074, China; MOE Key Laboratory for Biomedical Photonics, Collaborative Innovation Center for Biomedical Engineering, School of Engineering Sciences, Huazhong University of Science and Technology, Wuhan 430074, China; Department of Anatomy, Histology & Embryology, Fourth Military Medical University, Xi’an 710032, China; Key Laboratory of Molecular Biophysics of Ministry of Education, College of Life Science and Technology, Huazhong University of Science and Technology, Wuhan 430074, China; Department of Neurobiology, Affiliated Mental Health Center & Hangzhou Seventh People's Hospital, Zhejiang University School of Medicine, Hangzhou 310058, China; Liangzhu Laboratory, The MOE Frontier Research Center of Brain & Brain-machine Integration, State Key Laboratory of Brain-machine Intelligence, Hangzhou 311121, China

**Keywords:** high-fat diet, excessive daytime sleepiness, sleep–wake cycle, fragmented wakefulness, paraventricular thalamic nucleus, time-restricted feeding

## Abstract

High-fat diet (HFD)-induced obesity is a growing epidemic and major health concern. While excessive daytime sleepiness (EDS) is a common symptom of HFD-induced obesity, preliminary findings suggest that reduced wakefulness could be improved with time-restricted feeding (TRF). At present, however, the underlying neural mechanisms remain largely unknown. The paraventricular thalamic nucleus (PVT) plays a role in maintaining wakefulness. We found that chronic HFD impaired the activity of PVT neurons. Notably, inactivation of the PVT was sufficient to reduce and fragment wakefulness during the active phase in lean mice, similar to the sleep–wake alterations observed in obese mice with HFD-induced obesity. On the other hand, enhancing PVT neuronal activity consolidated wakefulness in mice with HFD-induced obesity. We observed that the fragmented wakefulness could be eliminated and reversed by TRF. Furthermore, TRF prevented the HFD-induced disruptions on synaptic transmission in the PVT, in a feeding duration-dependent manner. Collectively, our findings demonstrate that ad libitum access to a HFD results in inactivation of the PVT, which is critical to impaired nocturnal wakefulness and increased sleep, while TRF can prevent and reverse diet-induced PVT dysfunction and excessive sleepiness. We establish a link between TRF and neural activity, through which TRF can potentially serve as a lifestyle intervention against diet/obesity-related EDS.

## INTRODUCTION

Obesity, which has reached epidemic levels in many adults and children, occurs when energy intake chronically exceeds energy expenditure [1]. Obese patients often suffer from excessive daytime sleepiness (EDS), which has a significant impact on vigilance, concentration, attention and quality of life [2]. Excessive sleepiness during the active phase of the diurnal cycle is also reported in obese rodents, including genetically [3,4] and HFD-induced obese models [5]. Time-restricted feeding (TRF), which is known to alleviate metabolic disorders and restore clock gene oscillation in peripheral tissues of obese mice [6,7], can also improve obesity-induced irregularity in the sleep–wake cycle [8]. However, the underlying mechanisms remain largely obscure.

The paraventricular thalamic nucleus (PVT), which spans the entire rostrocaudal length of the midline thalamus, displays distinct functions across the anteroposterior axis [9]. It is associated with salience [10], arousal [11–13] and regulation of emotional [14,15] and motivational behaviors [16,17]. The PVT is also involved in the regulation of feeding behavior [18,19], with food intake further regulated by a complex interplay of circulating signals of energy homeostasis. Previous studies have found that the PVT is sensitive to energy balance. Hypoglycemia induces elevated activity of PVT-to-nucleus accumbens (NAc)-projecting neurons, resulting in orectic effects [20,21]. In contrast, administration of glucagon-like peptide-1 receptor (GLP-1R) agonists in the PVT reduces the activity of such neurons, resulting in anorectic effects [22].

Early studies in rats demonstrated that c-Fos (a marker of neuronal activity) increased in the PVT during the dark (active) phase compared to the light phase [23]. The PVT belongs to the thalamocortical arousal system. The PVT receives dense peptidergic fiber innervations from sleep–wake regulatory networks, including noradrenergic and serotonergic fibers from the brain stem, as well as histaminergic, orexinergic and neurotensin-containing fibers from the hypothalamus [24]. Widespread excitatory inputs to the PVT activate the cerebral cortex to cause wakefulness. However, PVT lesions can impair the integrity of wakefulness and induce sleepiness in humans and rodents [12,25].

Furthermore, recent studies have proposed that the PVT is also linked with arousal in cases that are independent of the light–dark cycle. In constant darkness, the level of c-Fos in the PVT at the time of scheduled access to a palatable meal is increased [26]. In addition, food entrainment changes the peak of daily oscillations of PER1 in the PVT [27]. Thus, we hypothesize that the activity of PVT neurons is critical to maintenance of wakefulness during the active phase of the diurnal cycle, which could be modulated by daily feeding schedules. As such, we applied TRF in the current study to prevent and reverse diet-induced PVT inactivity and fragmented wakefulness in the dark phase when mice are typically active [28].

We were able to recapitulate the EDS phenotype in mice with HFD-induced obesity. Chronic ad libitum (AL) HFD consumption shortened the duration of wakefulness and increased the fragmentation of wakefulness during the active phase. AL access to HFD decreased neuronal excitability and damaged the synaptic transmission of PVT, remodeling the excitation/inhibition (E/I) ratio. Consistently, inactivation of the PVT in lean mice reduced and fragmented wakefulness similar to HFD treatment, whereas restoration of PVT neuronal activity consolidated wakefulness in the obese mice. Moreover, this fragmented wakefulness could be prevented by active phase/night-time TRF. We found that active phase TRF also eliminated HFD-induced impairment on PVT synaptic activity, while treatment efficacy was dependent on feeding/starvation duration. Furthermore, TRF could not only prevent but also reverse the impact of HFD-induced obesity on the PVT and wakefulness. These results highlight the potential pathological mechanism underlying EDS associated with obesity and provide a non-pharmacological intervention against it.

## RESULTS

### AL HFD feeding impairs PVT neuronal activity

We subjected 6- to 7-week-old male wild-type C57BL/6J mice to a normal diet (ND) or an HFD under 24-hr AL feeding. After 8 weeks of feeding, compared to the ND group, the HFD mice showed prominent markers of obesity, including weight gain and elevated glucose tolerance (Supplementary Fig. S1A). Electroencephalogram–electromyogram (EEG–EMG) electrodes were implanted to monitor 24-hr sleep–wake stages in the ND and HFD mice (Supplementary Fig. S1B–D). HFD feeding induced a significant reduction in wakefulness and an increase in sleep (non-rapid eye movement (NREM) and rapid eye movement (REM) sleep) relative to ND during the active phase (Supplementary Fig. S1E–J), which appear to be the result of impaired wake maintenance as the wake bout duration was significantly decreased while the wake bout number was significantly increased during the active phase (Supplementary Fig. S1K and L). To further assess the effects of the feeding pattern on sleep, we analysed the frequency of microarousal (MA) events (MA count/NREM time), which is considered a reflection of sleep fragmentation. An MA event was defined as a brief awakening period (<5 s) during NREM sleep [29]. We found that HFD feeding increased the frequency of MA events during both the light and dark periods (Supplementary Fig. S1M). The distributions of episode duration show that HFD increased the short wakefulness episodes $( { <\! 30\,{\rm{s}}} )$ and decreased long duration $( { >\! 960\,{\rm{s}}} )$ and HFD also increased REM and NREM episodes for both short $( { <\! 30\,{\rm{s}}} )$ and long duration $( { >\! 30\,{\rm{s}}} )$ (Supplementary Fig. S1N). Consistently, HFD feeding increased ‘wake–NREM’ transitions during the dark phase and ‘NREM–REM’ transitions during both the light and dark phases (Supplementary Fig. S1O). Taken together, these results suggest that during the dark but not the light phase, there are increased wake episode counts accompanied by decreased wake episode duration in HFD mice. This fragmentation could in turn lead to increased sleep episodes during the dark phase, while sleep episode duration is not altered. We believe this phenomenon that occurs during the active phase of mice is similar to EDS in humans, which also occurs during the active phase. To explore the underlying mechanism of obesity-related EDS, we assessed the impact of HFD on multiple aspects of PVT activity. First, we recorded spike firing of PVT neurons in freely behaving mice and acute brain slices after AL HFD feeding.

**Figure 1.**
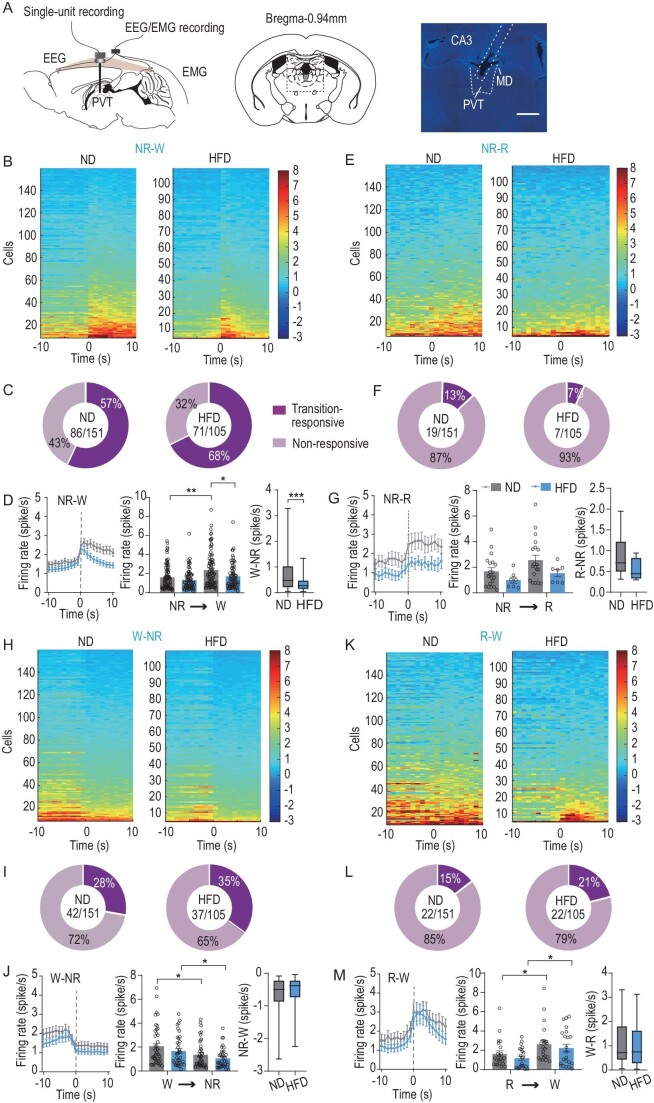
Sleep–wake transition-related firing in the PVT is disrupted by AL HFD feeding. (A) Schematic of experiment. Single-unit recording electrodes, EEG screw electrodes and EMG wires were implanted simultaneously (left). Coronal diagram of mouse brain (middle), representative image of electrode trace in the PVT (right). MD, mediodorsal thalamic nucleus. Scale bar, 500 μm. (B) Heat map showing average firing rates of neurons during state transitions from NREM to wakefulness. Neurons were sorted as mean firing rates 10 s after state transition (*n* = 7–9 mice, 105–151 units). (C) Pie chart depicting fraction of PVT neurons that respond to state transitions in ND (left) and HFD (right) mice. (D) Average firing rates of transition-related neurons during state transition period (left). Average firing rates 10 s before and after state transitions (middle). Box plots showing firing rates of wake–NREM in PVT neurons (right) in ND and HFD mice (*n* = 7–9 mice, 71–86 units, Mann–Whitney test). (E) Heat map showing average firing rates of neurons during state transitions from NREM to REM (*n* = 7–9 mice, 105–151 units). (F) Pie chart depicting fraction of PVT neurons that respond to state transitions in ND (left) and HFD (right) mice. (G) Average firing rates of transition-related neurons during state transition period (left). Average firing rates 10 s before and after state transitions (middle). Box plots showing firing rates of REM–NREM in PVT neurons (right) in ND and HFD mice (*n* = 7–9 mice, 7–19 units, Mann–Whitney test). (H) Heat map showing average firing rates of neurons during state transitions from wakefulness to NREM (*n* = 7–9 mice, 105–151 units). (I) Pie chart depicting fraction of PVT neurons that respond to state transitions in ND (left) and HFD (right) mice. (J) Average firing rates of transition-related neurons during state transition period (left). Average firing rates 10 s before and after state transitions (middle). Box plots showing firing rates of NREM–wake in PVT neurons (right) in ND and HFD mice (*n* = 7–9 mice, 37–42 units, Mann–Whitney test). (K) Heat map showing average firing rates of neurons during state transitions from REM to wakefulness. (L) Pie chart depicting fraction of PVT neurons that respond to state transitions in ND (left) and HFD (right) mice. (M) Average firing rates of transition-related neurons during state transition period (left). Average firing rates 10 s before and after state transitions (middle). Box plots showing firing rates of REM–wake in PVT neurons (right) in ND and HFD mice (*n* = 7–9 mice, 22 units, Mann–Whitney test). Data are means ± SEM. Box plots show median, quartiles (boxes) and range (whiskers). Dots represent individual experimental cells. *P < 0.05; **P < 0.01; ***P < 0.001.

To monitor PVT activity across the natural sleep–wake cycle, we implanted a movable 16-microwire bundle in the PVT for single-unit recordings and EEG–EMG electrodes for simultaneous polysomnography (Fig. 1A). In total, 151 and 105 well-isolated units were collected in the PVT of ND and HFD mice, respectively. To quantify the relative firing rates of the PVT neurons during different brain states, we plotted REM–NREM and wake–NREM modulations. Most neurons were in the first quadrant, including neurons with significantly higher firing rates during wakefulness and REM sleep than during NREM sleep. Intriguingly, HFD markedly reduced the REM-related firing rates (Supplementary Fig. S2A–C). The firing rates of PVT neurons during the different states were compared between ND and HFD mice. HFD feeding lowered the fraction of neurons with firing rates of >3 Hz and significantly decreased the mean firing rate of the PVT during REM sleep (Supplementary Fig. S2D–G). Hence, HFD impairs recurrent excitation of PVT neurons and thereby wake maintenance function.

To further characterize HFD-induced dysfunction, we compared PVT neuronal activity during different state transitions. Results showed that the HFD mice had markedly lower activity during the NREM-to-wake transition (Fig. 1B–D), as well as a decreasing trend in activity during the NREM-to-REM transition (Fig. 1E–G). However, HFD did not affect activity during the wake-to-NREM and REM-to-wake transitions (Fig. 1H and M). These results indicate that HFD decreases the excitability of PVT neurons and weakens their response to state transitions, both of which may impair maintenance of wakefulness.

For *in vitro* recordings, 500-ms depolarizing steps were used to assess the intrinsic properties of PVT neurons in slices of the ND- and HFD-treated mice (Supplementary Fig. S2H). HFD did not change the resting membrane potential or first spike latency (Supplementary Fig. S2I and K) but increased the spike threshold and rheobase of the PVT neurons (Supplementary Fig. S2J and L). These findings demonstrate that HFD reduces the intrinsic excitability of PVT neurons.

### AL HFD feeding impairs synaptic transmission and remodels E/I balance in PVT

We further investigated the PVT-related circuitry that is disrupted by HFD feeding. Cholera toxin subunit B (CTB) was injected into the NAc and bed nucleus of the stria terminalis (BNST), respectively, which are two known downstream targets of PVT projections [13]. After expression for 3 days, the miniature excitatory postsynaptic currents (mEPSCs) of NAc-projecting and BNST-projecting PVT neurons were tested in the presence of tetrodotoxin (TTX, 1 μM) by *in vitro* whole-cell recordings of PVT neurons (Supplementary Fig. S3A, B, G and H). The results showed that HFD feeding reduced the frequency of mEPSCs in both populations (Supplementary Fig. S3C, D, I and J). On the other hand, no difference was observed in amplitudes of mEPSCs between ND and HFD (Supplementary Fig. S3E, F, K and L). Therefore, HFD also impairs the excitatory synaptic transmission in the PVT, which may be induced by widespread deficits throughout the PVT.

Furthermore, 2 weeks of HFD feeding was sufficient to impair synaptic transmission in the PVT (Supplementary Fig. S4D–H), which was well before the onset of significant weight gain and elevated glucose tolerance (Supplementary Fig. S4A–C). This further supports the idea that the disruption of PVT synaptic transmission caused by HFD is not the result of obesity or altered glucose metabolism.

Then, outward miniature inhibitory postsynaptic currents (mIPSCs) were also examined. HFD significantly lowered the frequencies of mIPSCs but had no effect on the amplitude compared to ND (Supplementary Fig. S5A–C). We further tested the effect of HFD on presynaptic plasticity. An electron microscope was used to measure the number of synapses and postsynaptic density (PSD) size in the PVT of ND and HFD mice (Supplementary Fig. S5D). Fewer synapses were observed after HFD feeding, including asymmetric and symmetric synapses (Supplementary Fig. S5E–G). Moreover, HFD decreased the thickness and size of the PSD in the PVT synapses (Supplementary Fig. S5H–J). We also tested the spine density of AL-fed ND and HFD mice. AAV-CAG-EYFP was injected into the PVT and spines were analysed after EYFP expression. We found that many dendritic spines of PVT neurons were stubby, which is consistent with a previous study [30], and HFD decreased the spine density of PVT neurons (Supplementary Fig. S5K). We propose that HFD reduces synaptic transmission efficiency by decreasing the number of synapses and PSD size in the PVT.

We next performed extracellular stimulation to evoke excitatory postsynaptic currents (EPSCs), but no significant change was observed in the paired-pulse ratio of the PVT between HFD and ND mice (Supplementary Fig. S5L and M). These results indicate that AL HFD feeding does not impair synaptic transmission of the PVT by a presynaptic mechanism, but rather by decreasing synapse counts.

In addition, cells were held in voltage-clamp at different potentials (−70 and 10 mV) to separate evoked EPSCs from inhibitory postsynaptic currents (IPSCs) (Supplementary Fig. S5N). Varied amplitudes of evoked EPSCs and IPSCs were recorded in the PVT of ND and HFD mice. The evoked EPSC/IPSC amplitudes (E/I ratio) were lower in the PVT of HFD mice compared to ND mice (Supplementary Fig. S5O and P). Thus, these results indicate that AL HFD feeding remodels E/I balance in the PVT.

### Inactivation of PVT mimics the impact of AL HFD feeding on wakefulness

To explore whether PVT inactivation can mimic HFD-induced fragmented wakefulness, the tetanus neurotoxin (TeNT) [31], a protease to block neurotransmitter release by cleaving synaptobrevin-2, was employed to inactivate the PVT. A mixture of AAV-CAG-EGFP-2A-TeNT and AAV-CAG-ChR2-mCherry were injected into the PVT to test the efficiency of TeNT, and co-expression of EGFP and ChR2-mCherry in the same cells was observed. ChR2-mCherry-negative cells were examined using whole-cell patch-clamp recordings of the PVT in acute brain slices after AAV expression for 2 and 4 weeks (Supplementary Fig. 6A and B). Both the amplitudes and probabilities of light-evoked EPSCs decreased after TeNT expression for 4 weeks compared to that after 2 weeks (Supplementary Fig. S6C and D), indicating that PVT neurons expressing TeNT for 4 weeks are inactivated compared with PVT neurons expressing TeNT for 2 weeks.

**Figure 2.**
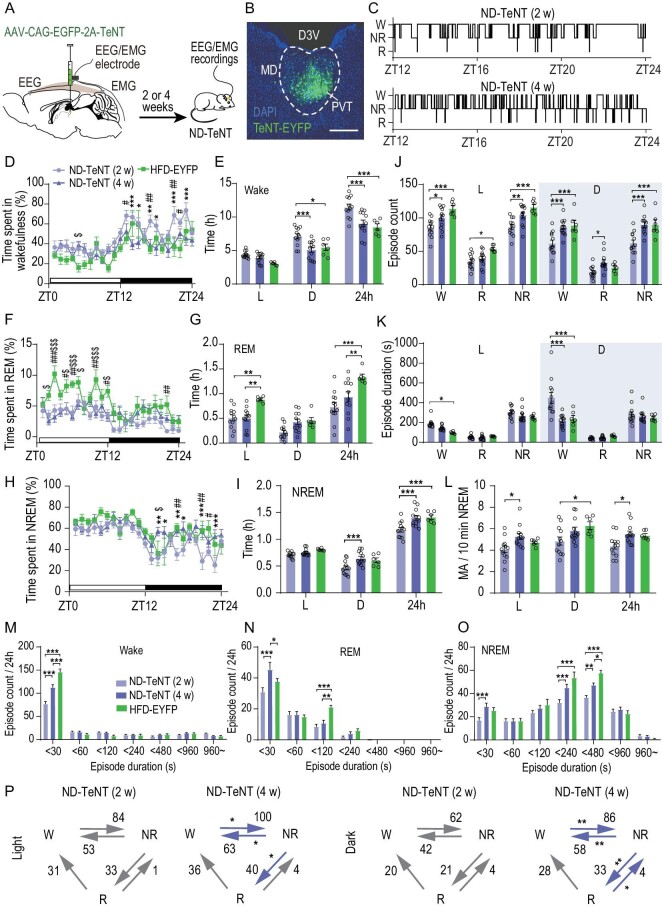
Inactivation of PVT mimics the impact of AL HFD feeding on wakefulness. (A) Schematic of virus injection and electrophysiological recording experiment. (B) Representative images showing expression of TeNT-EYFP in PVT. Scale bar, 300 μm. (C) Representative hypnograms of TeNT expression for 2 (top) and 4 weeks (bottom) in ND mice. (D) Hourly percentage of time spent in wakefulness for mice in three groups (TeNT expressed for 2 and 4 weeks in ND mice and EYFP expressed in HFD mice) (*n* = 6–12, two-way ANOVA, Bonferroni post hoc analysis). (E) Total time spent in wakefulness during 12-hr light (L, left), 12-hr dark (D, middle) or 24-hr LD cycle (24 h, right) in three groups (*n* = 6–12, two-way ANOVA, Bonferroni post hoc analysis). (F) Hourly percentage of time spent in REM for mice in three groups (*n* = 6–12, two-way ANOVA, Bonferroni post hoc analysis). (G) Total time spent in REM during 12-hr light (L, left), 12-hr dark (D, middle) or 24-hr LD cycle (24 h, right) in three groups (*n* = 6–12, two-way ANOVA, Bonferroni post hoc analysis). (H) Hourly percentage of time spent in NREM for mice in three groups (*n* = 6–12, two-way ANOVA, Bonferroni post hoc analysis). (I) Total time spent in NREM during 12-hr light (L, left), 12-hr dark (D, middle) or 24-hr LD cycle (24 h, right) in three groups (*n* = 6–12, two-way ANOVA, Bonferroni post hoc analysis). (J and K) Episode counts (J) and durations (K) of wakefulness (W), REM sleep (R) and NREM sleep (NR) during 12-hr light and 12-hr dark cycle in three groups (*n* = 6–12, two-way ANOVA, Bonferroni post hoc analysis). (L) Frequency of MA events during 12-hr light and 12-hr dark NREM sleep for mice in three groups (*n* = 6–12, two-way ANOVA, Bonferroni post hoc analysis). (M–O) Distributions of episode duration of wakefulness (M), REM sleep (N) and NREM sleep (O) stages. (P) Average number of transitions between vigilance states in ND mice after TeNT expression for 2 and 4 weeks during light (left) and dark phases (right) (*n* = 6–12, two-tailed paired *t*-test). Data are means ± SEM. Dots represent individual experimental animals. ‘W’ stands for wakefulness, ‘R’ stands for REM sleep and ‘N’ stands for NREM sleep. For (D), (F and H), significant differences between ND-TeNT (2 weeks) and ND-TeNT (4 weeks) are marked with ‘*’, differences between ND-TeNT (2 weeks) and HFD-EYFP are marked with ‘#’ and differences between ND-TeNT (4 weeks) and HFD-EYFP are marked with ‘$’. *P < 0.05; **P < 0.01; ***P < 0.001.

Next, we injected TeNT into the PVT of ND mice and recorded 24-hr EEG/EMG signals after 2 and 4 weeks of expression (Fig. 2A and B). EYFP was injected into the PVT of mice under chronic HFD feeding (HFD for 8 weeks) to serve as a control group. We were able to recapitulate EDS in obese individuals using this mouse model. Compared with 2 weeks of TeNT expression in the PVT, expression of 4 weeks led to a significant decrease in wakefulness and an increase in REM and NREM sleep during the dark phase, comparable with HFD-EYFP mice which served as a positive control (Fig. 2C–I). This was primarily due to shortened duration of wake episodes (Fig. 2J and K). TeNT expression for 4 weeks also increased the number of NREM episodes and frequency of MA events, similarly to HFD controls (Fig. 2L). The distributions of episode duration showed that inactivation of the PVT, like HFD, increased the short wakefulness episodes $( { <\! 30\,{\rm{s}}} )$ compared to the ND group (Fig. 2M–O). More ‘NREM–wake’ and ‘NREM–REM’ transitions were also observed after TeNT was expressed for 4 weeks in ND mice (Fig. 2P). There were no significant differences in sleep–wake duration and episodes in ND mice expressing TeNT (4 weeks) compared with HFD mice expressing EYFP in the PVT, although more REM sleep and short episodes were observed in HFD-EYFP mice. Taken together, these data demonstrate that inhibiting PVT neuronal activity leads to impaired wakefulness during the active phase, similar to that observed in HFD-fed mice.

### Activation of PVT neurons alleviates impaired wakefulness in obese mice

**Figure 3.**
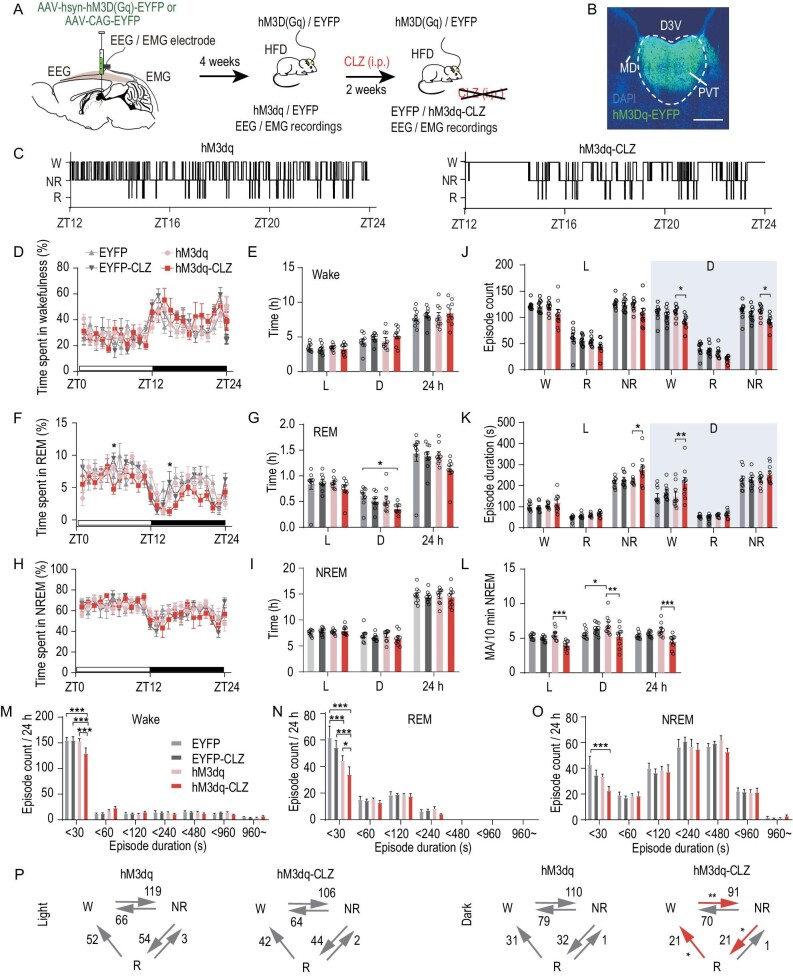
Activation of PVT neurons alleviates impairment of wakefulness in obese animals. (A) Schematic of virus injection and electrophysiological recording experiment. (B) Representative images showing expression of hM3Dq-EYFP in PVT. Scale bar, 300 μm. (C) Representative hypnograms of hM3Dq-expressing mice before (left) and after CLZ administration (right). (D) Hourly percentage of time spent in wakefulness for mice in four groups (before and after CLZ administration in hM3Dq- and EYFP-expressing mice) (*n* = 9, two-way ANOVA, Bonferroni post hoc analysis). (E) Total time spent in wakefulness during 12-hr light (L, left), 12-hr dark (D, middle) or 24-hr LD cycle (24 h, right) in four groups (*n* = 9, two-way ANOVA, Bonferroni post hoc analysis). (F) Hourly percentage of time spent in REM for mice in four groups (*n* = 9, two-way ANOVA, Bonferroni post hoc analysis). (G) Total time spent in REM during 12-hr light (L, left), 12-hr dark (D, middle) or 24-hr LD cycle (24 h, right) in four groups (*n* = 9, two-way ANOVA, Bonferroni post hoc analysis). (H) Hourly percentage of time spent in NREM for mice in four groups (*n* = 9, two-way ANOVA, Bonferroni post hoc analysis). (I) Total time spent in NREM during 12-hr light (L, left), 12-hr dark (D, middle) or 24-hr LD cycle (24 h, right) in four groups (*n* = 9, two-way ANOVA, Bonferroni post hoc analysis). (J and K) Episode counts (J) and durations (K) of wakefulness (W), REM sleep (R) and NREM sleep (NR) during 12-hr light and 12-hr dark cycle in four groups (*n* = 9, two-way ANOVA, Bonferroni post hoc analysis). (L) Frequency of MA events during 12-hr light and 12-hr dark NREM sleep for mice in four groups (*n* = 9, two-way ANOVA, Bonferroni post hoc analysis). (M–O) Distributions of episode duration of wakefulness (M), REM sleep (N) and NREM sleep (O) stages. (P) Average number of transitions between vigilance states in hM3Dq-expressing mice before and after CLZ administration during light (left) and dark phases (right) (*n* = 9, two-tailed paired *t*-test). Data are means ± SEM. Dots represent individual experimental animals. For (D), (F and H), significant differences between hm3Dq-CLZ and EYFP-CLZ are marked with ‘*’. *P < 0.05; **P < 0.01; ***P < 0.001.

We next tested whether increasing the activity of PVT neurons can rescue disrupted wakefulness following chronic HFD feeding (HFD for 8 weeks). We injected a virus containing a Gαq-coupled designer receptor exclusively activated by designer drug (DREADD) fused to the fluorescent protein EYFP (AAV2-hsyn-CAG-hM3Dq-EYFP) in the PVT of HFD-induced obese mice and injected AAV2-EF1a-CAG-EYFP to serve as the control. Clozapine (CLZ) was injected intraperitoneally to induce Gαq-mediated signal transduction and activate PVT neurons [32]. To validate the efficiency of the DREADD system *in vivo*, single-unit recordings were performed 4 weeks after virus injection. CLZ administration significantly increased the firing rates of PVT neurons (Supplementary Fig. S7A–E). In addition, CLZ increased the time spent in wakefulness and decreased the time spent in REM sleep 6 hr after CLZ injection in the hM3Dq-HFD group, but had no effect on the duration of wakefulness and sleep in the EYFP-HFD control group (Supplementary Fig. S7F–L). The PVT neurons of the HFD mice infected with hM3D were activated daily via an intraperitoneal injection of CLZ for 2 weeks and the animals were subjected to EEG/EMG recordings before and after CLZ treatment (Fig. 3A and B). CLZ significantly lengthened wake episode duration and reduced wake and NREM sleep episode number during the dark phase, indicative of a more consolidated wakefulness. CLZ also increased the duration of NREM sleep episodes during the light phase (Fig. 3J and K). The distributions of episode duration show that CLZ decreased the number of short episodes $( { <\! 30\,{\rm{s}}} )$ of wakefulness as well as REM and NREM episodes (Fig. 3M–O). Consistently with CLZ treatment leading to fewer wake and NREM sleep episodes during the dark phase, fewer transitions between vigilance states were observed during the dark phase (Fig. 3P). Moreover, CLZ treatment decreased the frequency of MA events (Fig. 3L). However, total durations of wakefulness and NREM sleep were not significantly altered by CLZ application, although REM sleep duration decreased (Fig. 3C–I). In the control group, CLZ treatment did not alter the sleep or wakefulness of EYFP-expressing mice.

Taken together, we found that activating PVT in animals on HFD consolidates nocturnal wakefulness. This is consistent with the effects of HFD and inactivation of PVT, which leads to fragmented wakefulness during the dark phase.

### TRF prevents HFD-induced impairment of nocturnal wakefulness

**Figure 4.**
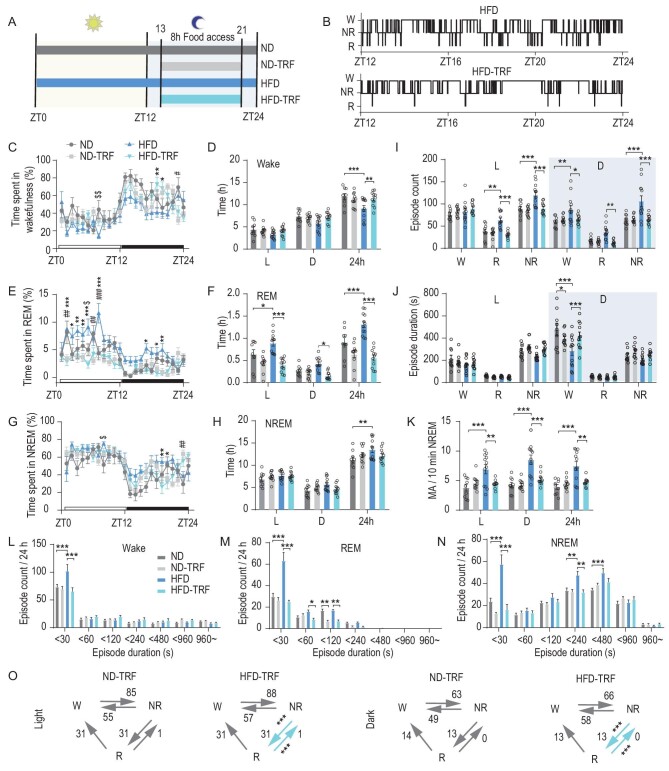
TRF prevents HFD-induced impairment of nocturnal wakefulness. (A) Experimental diagram showing feeding regimens. ND, ND AL feeding; ND–TRF, daily 8-hr ND time-restricted feeding; HFD, HFD AL feeding; HFD–TRF, daily 8-hr HFD time-restricted feeding. HFD–TRF and ND–TRF groups could access food from ZT13 to ZT21. (B) Representative hypnograms of HFD (top) and HFD–TRF (bottom) mice. (C) Hourly percentage of time spent in wakefulness for mice in four groups (ND, HFD, ND–TRF and HFD–TRF) (*n* = 8–10, two-way ANOVA, Bonferroni post hoc analysis). (D) Total time spent in wakefulness during 12-hr light (L, left), 12-hr dark (D, middle) or 24-hr LD cycle (24 h, right) in four groups (*n* = 9–12, two-way ANOVA, Bonferroni post hoc analysis). (E) Hourly percentage of time spent in REM for mice in four groups (*n* = 9–12, two-way ANOVA, Bonferroni post hoc analysis). (F) Total time spent in REM during 12-hr light (L, left), 12-hr dark (D, middle) or 24-hr LD cycle (24 h, right) in four groups (*n* = 9–12, two-way ANOVA, Bonferroni post hoc analysis). (G) Hourly percentage of time spent in NREM for mice in four groups (*n* = 9–12, two-way ANOVA, Bonferroni post hoc analysis). (H) Total time spent in NREM during 12-hr light (L, left), 12-hr dark (D, middle) or 24-hr LD cycle (24 h, right) in four groups (*n* = 9–12, two-way ANOVA, Bonferroni post hoc analysis). (I and J) Episode counts (I) and durations (J) of wakefulness (W), REM sleep (R) and NREM sleep (NR) states (*n* = 9–12, two-way ANOVA, Bonferroni post hoc analysis). (K) Representative recording of MA event during NREM sleep (left). Frequency of MA events (right) (*n* = 9–12, two-way ANOVA, Bonferroni post hoc analysis). (L–N) Distributions of episode duration of wakefulness (L), REM sleep (M) and NREM sleep (N) stages (*n* = 9–12, two-way ANOVA, Bonferroni post hoc analysis). (O) Average number of transitions between vigilance states in ND–TRF and HFD–TRF mice during light (left) and dark phases (right) (*n* = 9–12, two-tailed unpaired *t*-test). Data are means ± SEM. Dots represent individual experimental animals. The ND and HFD data here are the same as those in Supplementary Fig. S1. For (C), (E), (G) and (O), significant differences between HFD and HFD–TRF are marked with ‘*’, differences between ND and ND–TRF are marked with ‘#’ and differences between HFD and ND are marked with ‘$’. *P < 0.05; **P < 0.01; ***P < 0.001 as indicated.

TRF is effective at preventing obesity and other metabolic disruptions associated with HFD [6]. To test whether TRF is also effective at preventing HFD-induced EDS, we subjected mice to a ND or an HFD under 24-hr AL feeding or time-restricted access to food only during their natural nocturnal feeding time (ND–TRF; HFD–TRF) in the same environment (Fig. 4A). EEG–EMG electrodes were implanted to monitor 24-hr sleep–wake stages in mice (Fig. 4B). TRF prevented HFD-induced reduction in wakefulness (Fig. 4C and D). In addition, HFD feeding increased sleep (NREM and REM sleep) total time (Fig. 4E–H). Notably, TRF protected against the HFD-induced fragmented and reduced wakefulness during the dark/active phase (Fig. 4I and J). We found that TRF could eliminate the HFD-induced increase in MA events during both the light and dark periods (Fig. 4K). The distributions of episode duration show that HFD increased the short wakefulness episodes $( { <\! 30\,{\rm{s}}} )$, and also REM and NREM episodes for both short $( { <\! 30\,{\rm{s}}} )$ and long duration $( { >\! 30\,{\rm{s}}} )$. TRF feeding could reverse this increase in sleep and wake episodes (Fig. 4L–N). For the increased ‘wake–NREM’ transitions during the dark phase and ‘NREM–REM’ transitions during both the light and dark phases of HFD, the ‘NREM–REM’ transitions became normal under TRF, and the ‘wake–NREM’ transitions also showed a trend of decrease (Fig. 4O and Supplementary Fig. S1O). Therefore, these results suggest that TRF could prevent HFD-induced impaired wake maintenance during the active phase, including eliminating the HFD-induced reduced wake episode duration and increased episode.

To further explore the effects of HFD and TRF on sleep homeostasis and circadian rhythm, two processes that regulate sleep [33], we analysed EEG delta power and rebound sleep following sleep deprivation that are believed to be regulated by sleep homeostasis, as well as the daily pattern of food intake and wheel-running rhythm. We found that during baseline sleep, AL HFD feeding did not affect NREM EEG delta power, while TRF regimens increased the EEG delta power in both the light and the dark phase (Supplementary Fig. S8A), suggesting that HFD did not alter sleep pressure while TRF enhanced sleep pressure.

Next, we examined the homeostatic responses to sleep deprivation. Mice were subjected to 6 hr of continuous sleep deprivation. Both groups showed a rebound in NREM and REM sleep during the recovery period, including the remaining 6 hr of the light phase (L2) and the subsequent 12 hr of the dark phase (D1 and D2) (Supplementary Fig. S8B and C). During the first half of the dark phase (D1), HFD-fed mice showed less NREM rebound sleep compared with ND and HFD–TRF mice, while no significant difference was observed for REM rebound sleep. HFD mice displayed reduced recovery sleep, probably because they have increased baseline sleep during the D1 period (Supplementary Fig. S8D and E). In addition, no significant change in NREM delta or REM theta power density was found in the four groups after sleep deprivation (Supplementary Fig. S8F). These results demonstrate that the AL HFD feeding leads to reduced sleep rebound, which is reversed by TRF.

Finally, circadian rhythms of the four groups were examined. AL HFD mice showed decreased food intake during the night (ZT12–18) and increased food intake during the day (ZT0–12). TRF regimens restricted the food access to 8 hr, which eliminated the alteration of temporal food-intake pattern induced by HFD feeding (Supplementary Fig. S8G). In addition, AL HFD feeding mice exhibited no remarkable difference in wheel-running behavior, periodogram power or phase compared with ND and HFD–TRF groups (Supplementary Fig. S8H–K). These results indicate that HFD leads to reduced sleep homeostasis and altered daily pattern of food intake, which can be corrected by TRF.

### TRF prevents HFD-induced impairment of PVT synaptic transmission in a feeding duration-dependent manner

**Figure 5.**
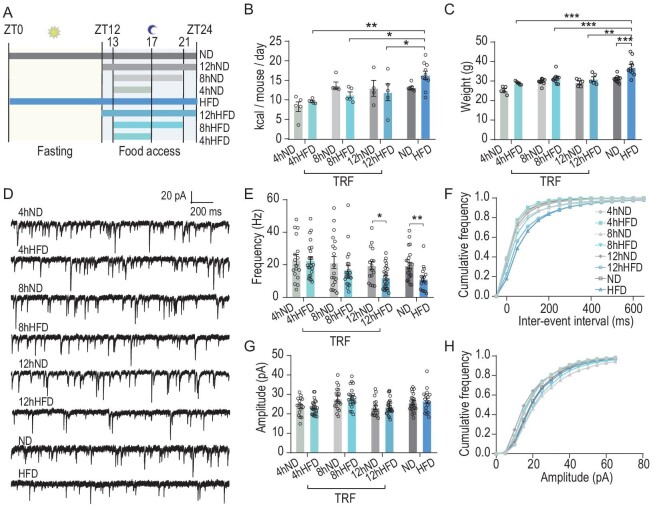
TRF prevents HFD-induced impairment of PVT synaptic transmission in a feeding duration-dependent manner. (A) Experimental diagram showing feeding regimens. ND, ND AL feeding; 12hND, daily 12-hr ND time-restricted feeding; 8hND, daily 8-hr ND time-restricted feeding; 4hND, daily 4-hr ND time-restricted feeding; HFD, HFD AL feeding; 12hHFD, daily 12-hr HFD time-restricted feeding; 8hHFD, daily 8-hr HFD time-restricted feeding; 4hHFD, daily 4-hr HFD time-restricted feeding. Mice in all groups were fed for 8 weeks. (B and C) Daily energy intake (B) and mean weight (C) of groups after feeding processes in (A) for 8 weeks (*n* = 4–10, two-way ANOVA, Bonferroni post hoc analysis). (D) Representative mEPSC traces recorded from PVT neurons for groups after feeding processes in (A) for 8 weeks. (E and F) Mean frequency (E) and cumulative fraction curves of inter-event intervals (F) of mEPSCs (*n* = 3–5 mice, 16–25 cells, Mann–Whitney test). (G and H) Mean amplitude (H) and cumulative fraction curves of amplitudes (G) of mEPSCs (*n* = 3–5 mice,16–25 cells, Mann–Whitney test). Data are means ± SEM. For (B) and (C), dots represent individual experimental animals. For (E–H), dots represent individual experimental cells. *P < 0.05; **P < 0.01; ***P < 0.001 as indicated.

To explore the underlying mechanism of the protective effects of TRF on HFD-induced nocturnal fragmented wakefulness, we assessed the impact of different feeding patterns on PVT synaptic activity. Mice were subjected to a night-time HFD feeding paradigm for 8 weeks, with food access for 4, 8 or 12 hr (Fig. 5A). Food/calorie consumption was comparable among the TRF groups but was lower than that in the HFD group. For each feeding condition, no differences in calorie intake were observed between the ND and HFD mice (Fig. 5B). Under the 8- and 12-hr TRF conditions, the bodyweights of the ND and HFD mice were also comparable (Fig. 5C). Then, mEPSCs were examined by *in vitro* whole-cell recordings of PVT neurons. Interestingly, there was no difference in the frequency of mEPSCs in the PVT of ND and HFD mice under 4- and 8-hr TRF, but mEPSC frequency was significantly reduced in the HFD mice under 12-hr TRF, similar to that found under AL (Fig. 5D–F). The mEPSC amplitudes under various TRF schedules did not significantly differ between the HFD and ND groups (Fig. 5G and H). Hence, a TRF paradigm with food access for ≤8-hr can prevent the detrimental effects of a HFD on PVT synaptic transmission. It is worth noting that under 8- and 12-hr TRF, calorie intake and weight were similar between the HFD and ND mice, and reduced mEPSC frequency was only observed in the 12-hr TRF HFD group. This strongly implicates that HFD-induced impairment of PVT synaptic transmission is not caused by obesity or major perturbation of metabolic homeostasis but by food content and feeding duration.

### TRF can reverse HFD-induced impairment of PVT synaptic transmission and nocturnal wakefulness

**Figure 6.**
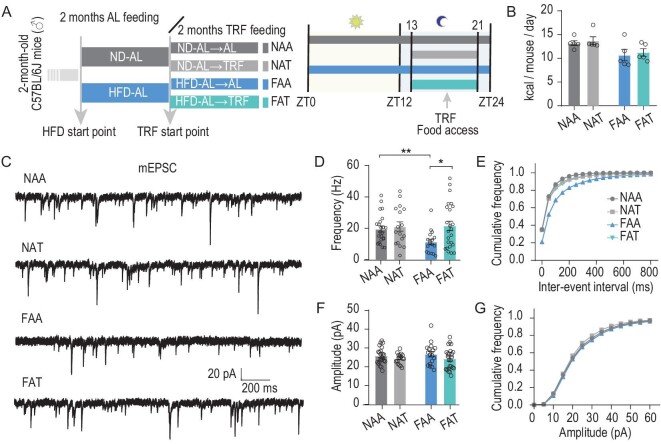
TRF reverses HFD-induced impairment of PVT synaptic transmission. (A) Experimental diagram showing study feeding regimens. NAA, ND AL feeding for 4 months; NAT, ND AL feeding for 2 months, then daily 8-hr ND time-restricted feeding for 2 months; FAA, HFD AL feeding for 4 months; FAT, HFD AL feeding for 2 months, then daily 8-hr HFD time-restricted feeding for 2 months. (B) Average daily energy intake of groups treated with different feeding paradigms in (A) (*n* = 5, two-way ANOVA, Bonferroni post hoc analysis). (C) Representative mEPSC traces recorded from PVT neurons for groups after feeding processes in (A). (D and E) Mean frequency (D) and cumulative fraction curves of inter-event intervals (E) of mEPSCs (*n* = 3–5 mice, 17–24 cells, Mann–Whitney test). (F and G) Mean frequency (F) and cumulative fraction curves of inter-event intervals (G) of mEPSCs (*n* = 3–5 mice, 17–24 cells, Mann–Whitney test). Data are means ± SEM. For (B), dots represent individual experimental animals. For (D–G), dots represent individual experimental cells. The FAA and NAA data here are the same as those in Fig. 2 HFD and ND. *P < 0.05 as indicated.

As 8 hr of TRF is sufficient to prevent the reduction in PVT synaptic transmission and fragmented wakefulness caused by HFD, we next tested whether this feeding paradigm is also effective at reversing these impairments under chronic HFD. Thus, mice were fed an AL ND or HFD for 2 months, then maintained at AL (ND AL feeding for 4 months [NAA], HFD AL feeding for 4 months [FAA]) or switched to TRF (ND AL feeding for 2 months, then TRF for 2 months [NAT], HFD AL feeding for 2 months, then TRF for 2 months [FAT]) (Fig. 6A) for another 2 months. We tested the food consumption of FAA and FAT mice. No difference was observed between the FAA and FAT groups (Fig. 6B). mEPSCs were examined to quantify the synaptic activity of PVT neurons. We observed lower frequency of mEPSCs in FAA relative to NAA. FAT significantly elevated the frequencies of mEPSCs but had no effect on amplitude compared to FAA (Fig. 6C–G). Therefore, TRF could rescue HFD-induced impairment of PVT activity and wakefulness.

**Figure 7.**
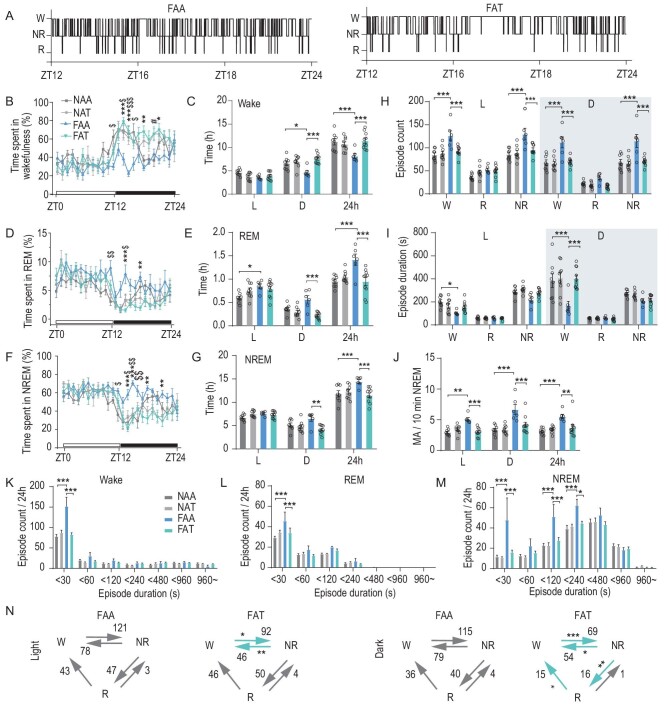
TRF reverses HFD-induced impairment of nocturnal wakefulness. (A) Representative hypnograms of FAA (left) and FAT (right) mice. (B) Hourly percentage of time spent in wakefulness for mice in four groups (NAA, FAA, NAT and FAT) (*n* = 6–10, two-way ANOVA, Bonferroni post hoc analysis). (C) Total time spent in wakefulness during 12-hr light (L, left), 12-hr dark (D, middle) or 24-hr LD cycle (24 h, right) in four groups (*n* = 6–10, two-way ANOVA, Bonferroni post hoc analysis). (D) Hourly percentage of time spent in REM for mice in four groups (*n* = 6–10, two-way ANOVA, Bonferroni post hoc analysis). (E) Total time spent in REM during 12-hr light (L, left), 12-hr dark (D, middle) or 24-hr LD cycle (24 h, right) in four groups (*n* = 6–10, two-way ANOVA, Bonferroni post hoc analysis). (F) Hourly percentage of time spent in NREM for mice in four groups (*n* = 6–10, two-way ANOVA, Bonferroni post hoc analysis). (G) Total time spent in NREM during 12-hr light (L, left), 12-hr dark (D, middle) or 24-hr LD cycle (24 h, right) in four groups (*n* = 6–10, two-way ANOVA, Bonferroni post hoc analysis). (H and I) Episode counts (H) and durations (I) of wakefulness (W), REM sleep (R) and NREM sleep (NR) states (*n* = 6–10, two-way ANOVA, Bonferroni post hoc analysis). (J) Frequency of MA events (*n* = 6–10, two-way ANOVA, Bonferroni post hoc analysis). (K–M) Distributions of episode duration of wakefulness (K), REM sleep (L) and NREM sleep (M) stages (*n* = 6–10, two-way ANOVA, Bonferroni post hoc analysis). (N) Average number of transitions between vigilance states in FAA and FAT mice during light (left) and dark phases (right) (*n* = 6–10, two-tailed unpaired *t*-test). Data are means ± SEM. Dots represent individual experimental animals. For (B), (D and F), significant differences between FAA and FAT are marked with ‘*’, differences between NAA and NAT are marked with ‘#’ and differences between FAA and NAA are marked with ‘$’. *P < 0.05; **P < 0.01; ***P < 0.001 as indicated.

Based on 24-hr EEG/EMG recordings, 8-hr TRF rescued the reduction in wake duration and fragmentation of wakefulness caused by HFD (Fig. 7B, C, H, I and K). This was accompanied by a reduction of sleep duration, number of sleep episodes, MA frequency and vigilance transitions (Fig. 7D–G, J, L and M). In short, an 8-hr TRF schedule rescues the reduction in wakefulness and excessive sleep elicited by HFD.

## DISCUSSION

Many studies on the health consequences of obesity have focused on cardiovascular and metabolic diseases, with little known about EDS associated with obesity. EDS is a highly prevalent condition in obese patients and can impact personal and occupational safety. EDS contributes to motor vehicle accidents [34] and the risk of medical errors [35]. Furthermore, EDS is associated with mental health disorders, such as depression and anxiety [36]. Therefore, restoring normal wakefulness and reducing EDS are critical for overall health, daytime performance and work safety. Obstructive sleep apnea (OSA) is a known cause of both EDS and fragmented sleep, especially in the obese population, but mounting evidence indicates that EDS occurs in obesity independently of OSA [2]. In addition, studies suggest there is no correlation between bodyweight and EDS [5]. Excess nutrients can also induce drowsiness [37], which has led to the hypothesis that chronic positive energy balance, not excessive adiposity, is the primary contributor to EDS. In the current study, we showed that HFD feeding for 2 weeks reduced the frequency of mEPSCs in the PVT but did not alter bodyweight or blood glucose (Supplementary Fig. S4A–H). Previous research has also observed excessive sleepiness during the active phase after HFD feeding for 2 weeks [38]. These results highlight the effects of chronic positive energy balance on the PVT and its underlying role in HFD-induced EDS.

TRF is a well-accepted strategy for improving metabolic and cardiovascular health, and even for extending the life span in various animal models and humans [6,39,40]. The influence of TRF on the brain, however, remains poorly characterized. Although TRF can increase wakefulness during the active phase and enhance sleep duration and quality during the rest phase in both fruit flies and mice, the relevant mechanism is not clear [41,42]. Our findings demonstrated the protective role of TRF against the HFD-induced decrease in PVT synaptic transmission, which eliminated the impact of HFD on wakefulness and excessive sleepiness. This effect was clearly not a consequence of changes in food intake, bodyweight or blood glucose, and the threshold for effective TRF ranged from 8 to 12 hr of feeding per day. Remarkably, previous studies on mice have shown that limiting daily high-fat and high-sugar intake to 8–12 hr can prevent diet-induced obesity and metabolic disorders, although calorie intake does not differ from AL feeding [39,43]. In addition, the shorter the daily feeding duration, the better the preventive effects [43], similar to the effects of TRF on PVT neural activities. Given the importance of sleep/wakefulness in modulating physiological homeostasis [44], it is reasonable to suspect that the impact of TRF on metabolic and cardiovascular function may also be mediated, in part, by optimized sleep/wakefulness.

Sleep is thought to be regulated by homeostatic and circadian processes [33]. Here, we observed that HFD had no effect on NREM delta power, indicating that HFD did not change sleep homeostasis under baseline. However, TRF regimens increased the EEG power (Supplementary Fig. S8A). Many studies have revealed that sleep slow wave activity (SWA) is positively correlated with not only wake duration, but also wake ‘intensity’; for example, the SWA after exploring and learning is higher than that after automatic behaviors [45]. Hence, we speculate TRF may enhance the wake intensity during the active phase, and thus increase sleep pressure and the depth of NREM sleep. Furthermore, we also employed a sleep-deprivation paradigm to probe the homeostatic process. HFD-fed animals showed reduced rebound sleep, indicative of reduced sleep need. This means the impaired wakefulness observed in these animals cannot be attributed to the alteration of sleep need, as reduced sleep need cannot explain the fragmented wakefulness. This is consistent with the notion that HFD impairs nocturnal wake but not sleep.

On the other hand, we cannot rule out the possibility that HFD impairs wakefulness by disrupting the circadian rhythm as HFD is known to disturb the circadian rhythm [46]. Further investigations will be required to characterize the role of circadian disruption in HFD-induced fragmentation of wakefulness. In addition, we agree that some of the effects of TRF on sleep/wakefulness are due to an engagement of feeding and locomotion during the dark period, which is accompanied by increased arousal. Indeed, the health-promoting effects of TRF arise from eating and being active at the right time of the day. However, we believe this cannot quite explain the protective effects of TRF on synaptic transmission of PVT, which were actually measured during the light period. Given that PVT is known to play an important role in maintaining wakefulness, we believe at least some of the effects of TRF on sleep/wakefulness is mediated by the PVT. This is supported by the data showing that activating PVT via the chemo-genetic method can increase wake bout duration and decrease sleep during the dark phase in HFD-fed animals (Fig. 3).

Our findings demonstrate that AL access to HFD results in inactivation of the PVT, which impaired nocturnal wakefulness and induced excessive sleepiness. TRF, as an intervention for HFD feeding, could effectively prevent and reverse the HFD-induced decreased PVT synaptic transmission and wake impairments, but the mechanism underlying how TRF affects synaptic transmission of PVT is yet unclear. Environmental light provides the principal entrainment signal to the molecular clock in the suprachiasmatic nucleus (SCN) and produces synchronized rhythms of behavior and physiology. However, the molecular clocks are also present in numerous tissues, including the liver, kidney, lung and heart, and the phases of these clocks are distinct from that of the SCN [47]. Besides the light–dark cycle, the feeding–fasting cycle is another major entrainment signal for the circadian clock, which acts independently of the SCN. A large body of evidence demonstrates that TRF is a strong zeitgeber of peripheral clocks and TRF restores clock gene oscillation in peripheral tissues of obese animals [7].

In the current study, AL HFD feeding resulted in a dampening of the food-intake rhythm, which may impair the sleep–wake cycle, while TRF imposed rhythmic food intake (Supplementary Fig. S8G). Therefore, we speculate that the effects of TRF on PVT may occur by preventing a disrupted gut clock. AL HFD feeding induces a positive metabolic state and obesity, and damages the rhythmic synthesis/release of these hormones in the circulation, including ghrelin, leptin, melanocyte-stimulating hormone (α-MSH) and endocannabinoids. In addition, obesity induces hormonal resistance. The arcuate nucleus (ARC) is a major site of ghrelin and leptin sensing, and disruption of hormones may affect the activity of the ARC [48]. The PVT receives input from the ARC, which may contribute to HFD-induced wake/sleep disorders [18]. On the other hand, obesity induces an activation of the endocannabinoid system (ECS) and increased concentrations of endocannabinoids in the circulation [49]. A previous report showed that intracellular cannabinoid receptors can modulate low-threshold spike (LTS)-induced slow afterdepolarization (sADP) in the PVT [50]. TRF can resume rhythmic feeding events, which in turn may re-establish the rhythms of these gut–brain axis-related hormones. Hence, TRF may rescue the HFD-induced decrease in synaptic transmission of the PVT by restoring the rhythmic release of gut hormones. Additionally, recent studies have shown that PVT can be entrained by food. TRF changes the daily oscillations of PER1 and c-Fos expression in the PVT [26,27]. We also speculate that HFD may disrupt the clock in the PVT, while TRF may directly restore clock gene oscillation in the PVT.

In summary, we identified reduced PVT synaptic transmission as an underlying mechanism leading to the reduction in wakefulness and excessive sleep caused by AL HFD feeding. Importantly, we found that TRF prevented and reversed the HFD-induced impairment of PVT neural activity and wakefulness. As TRF has shown promising effects on improving metabolic indices in obese people, we believe this strategy may alleviate EDS associated with obesity and diet.

## MATERIALS AND METHODS

Detailed materials and methods are available in the Supplementary data.

## Supplementary Material

nwac222_Supplemental_Files
