# Supplementary material for: Time-restricted feeding is an intervention against excessive dark-phase sleepiness induced by obesogenic diet
Source: Natl Sci Rev. 2022 Oct 16;10(1):nwac222. doi: 10.1093/nsr/nwac222 (PMC9942665; doi:10.1093/nsr/nwac222)
Supplement: nwac222_Supplemental_Files [file nwac222_supplemental_files.zip › Supplementary_data.docx]

**Figure S1 related to Figure 1**


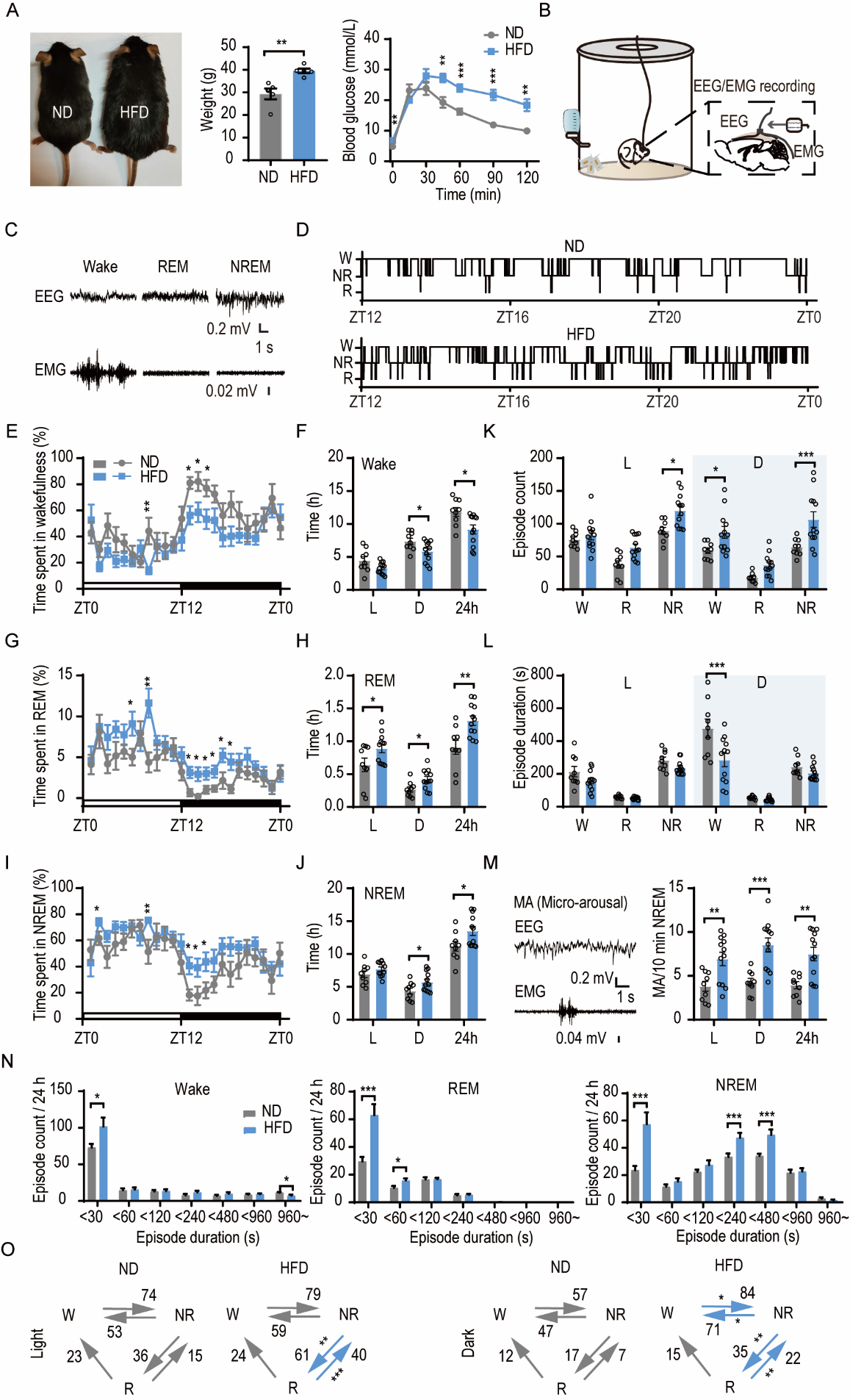


**Figure S1. AL HFD feeding impairs of nocturnal wakefulness.**

(A) Photo of representative ND and HFD mice (left). Mean weight (middle) and blood glucose levels in glucose tolerance test (right, GTT) of ND and HFD mice (n = 4, two-tailed unpaired *t* test).

(B) Schematic of experimental setup to EEG/EMG recording.

(C) Representative EEG/EMG traces at distinct states. EEG scalebar Y-axis: 0.2 mV, X-axis: 1 s. EMG scalebar Y-axis: 0.02 mV.

(D) Representative hypnograms of ND (top) and HFD (bottom) mice.

(E) Hourly percentage of time spent in wakefulness for ND and HFD mice (n = 9–12, two-tailed unpaired *t* test).

(F) Total time spent in wakefulness during 12-hr light (L, left), 12-hr dark (D, middle), or 24-hr LD cycle (24h, right) for ND and HFD mice (n = 9–12, two-tailed unpaired *t* test).

(G) Hourly percentage of time spent in REM for ND and HFD mice (n = 9–12, two-tailed unpaired *t* test).

(H) Total time spent in REM during 12-hr light (L, left), 12-hr dark (D, middle), or 24-hr LD cycle (24h, right) for ND and HFD mice (n = 9–12, two-tailed unpaired *t* test).

(I) Hourly percentage of time spent in NREM for ND and HFD mice (n = 9–12, two-tailed unpaired *t* test).

(J) Total time spent in NREM during 12-hr light (L, left), 12-hr dark (D, middle), or 24-hr LD cycle (24h, right) for ND and HFD mice (n = 9–12, two-tailed unpaired *t* test).

(K-L) Episode counts (K) and durations (L) of wakefulness (W), REM sleep (R), and NREM sleep (NR) states (n = 9–12, two-tailed unpaired *t* test).

(M) Representative recording of MA event during NREM sleep (left). Frequency of MA events (right) (n = 9–12, two-tailed unpaired *t* test).

(N) Distributions of episode duration of wakefulness (left), REM sleep (middle), and NREM sleep (right) stages (n = 9–12, two-tailed unpaired *t* test).

(O) Average number of transitions between vigilance states in ND and HFD mice during light (left) and dark phases (right) (n = 9–12, two-tailed unpaired *t* test).

Data are means ± SEM. Dots represent individual experimental animals. “W” stands for wakefulness, “R” stands for REM sleep, and “N” stands for NREM sleep. **p* < 0.05; ***p* < 0.01; ****p* < 0.001 as indicated.

**Figure S2 related to Figure 1**


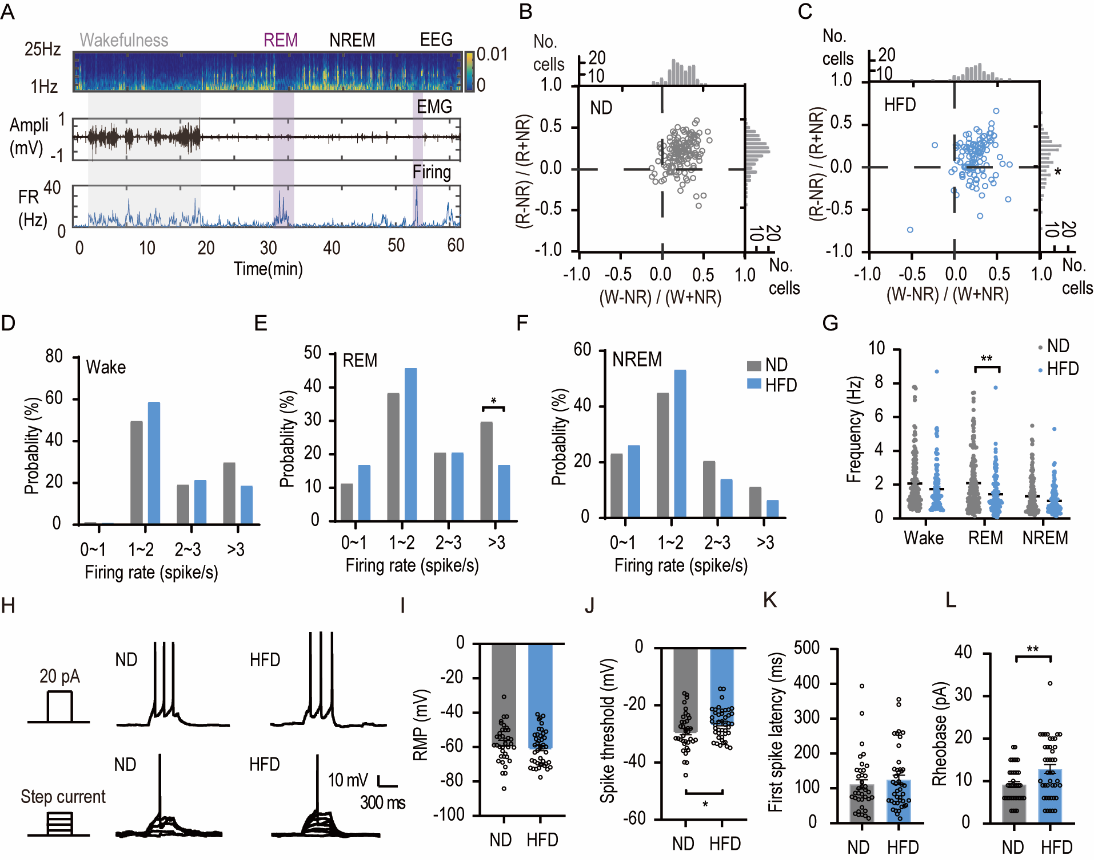


**Figure S2. AL HFD feeding impairs excitability of PVT neurons.**

(A) Firing rates of example neuron in PVT over 60 min. Brain states are colored (wakefulness, gray shading; REM, purple shading; NREM, white).

(B-C) Summary of firing-rate modulation of PVT cells in ND (B) and HFD (C) mice. (ND, n = 9 mice,151 units; HFD, n = 7 mice,105 units, Kolmogorov-Smirnov test).

(D-F) Histogram of firing rates of PVT cells during wakefulness (D), REM sleep (E), and NREM sleep (F) (ND, n = 9 mice,151 units; HFD, n = 7 mice,105 units, Chi-square test).

(G) Mean firing rate of PVT neurons during different brain states in ND and HFD mice (ND, n = 9 mice,151 units; HFD, n = 7 mice,105 units, Mann-Whitney test).

(H) Representative traces of voltage responses to 500-ms step current injection in current-clamp whole-cell configurations of PVT neurons.

(I-L) Analysis of intrinsic electrophysiological properties, including resting membrane potential (RMP) (I), spike threshold (J), first-spike latency (K), and rheobase current (L) (5–6 mice, 37–43 cells, two-tailed unpaired *t* test and Mann-Whitney test).

Data are means ± SEM. Dots represent individual experimental cells. **p* < 0.05; ***p* < 0.01; ****p* < 0.001 as indicated.

**Figure S3 related to Figure 1**


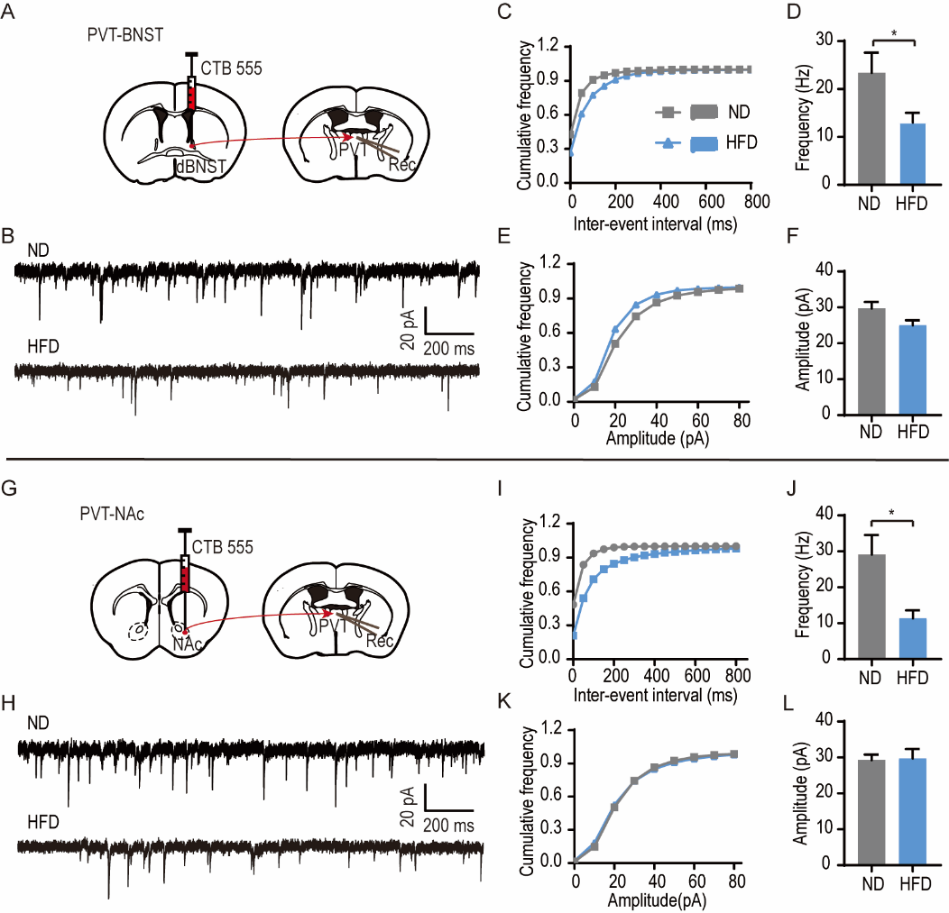


**Figure S3. HFD feeding decreases the synaptic transmission of BNST and NAc- projecting PVT neurons.**

(A, G) Diagram of experimental design.

(B, H) Representative mEPSC traces recorded from BNST- (B) and NAc- (H) projecting PVT neurons for ND and HFD mice.

(C, D) Cumulative fraction curves of inter-event intervals (C) and mean frequency (D) of mEPSCs of BNST-projecting PVT neurons (n = 5-9 cells, Mann-Whitney test).

(E, F) Cumulative fraction curves of amplitudes (E) and mean amplitude (F) of mEPSCs of BNST-projecting PVT neurons (n = 5-9 cells, Mann-Whitney test).

(I, J) Cumulative fraction curves of inter-event intervals (I) and mean frequency (J) of mEPSCs of NAc-projecting PVT neurons (n = 5-6 cells, Mann-Whitney test).

(K, L) Cumulative fraction curves of amplitudes (K) and mean amplitude (L) of mEPSCs of NAc-projecting PVT neurons (n = 5-6 cells, Mann-Whitney test).

Data are means ± SEM. **p* < 0.05 as indicated.

**Figure S4 related to Figure 1**


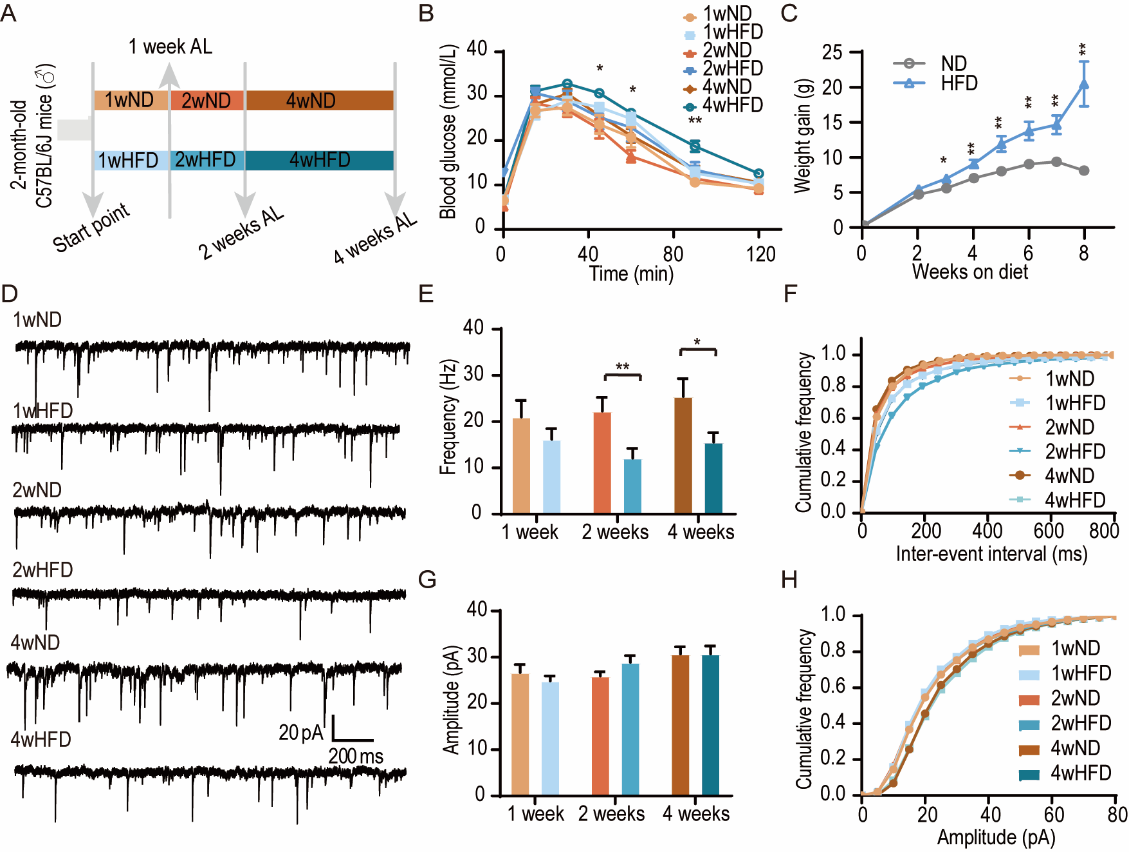


**Figure S4. Time course of HFD-induced impairment in PVT synaptic transmission.**

(A) Schematic of study experiment.

(B-C) Blood glucose levels during GTT (B). Mean weights of mice during ND and HFD feeding (C) (n = 5–10, two-tailed unpaired *t* test).

(D) Representative mEPSC traces from PVT neurons at different levels in ND and HFD mice (1-week, 2-weeks, and 4-weeks of ND and HFD).

(E, G) Mean frequency (E) and amplitude (G) of mEPSCs from PVT neurons in different groups.

(F, H) Cumulative fraction curves of inter-event intervals (F) and amplitudes (H) of mEPSCs from PVT neurons in different groups (n = 3–4 mice,14–24 cells, Mann Whitney test).

Data are means ± SEM. **p* < 0.05; ***p* < 0.01.

**Figure S5 related to Figure 1**


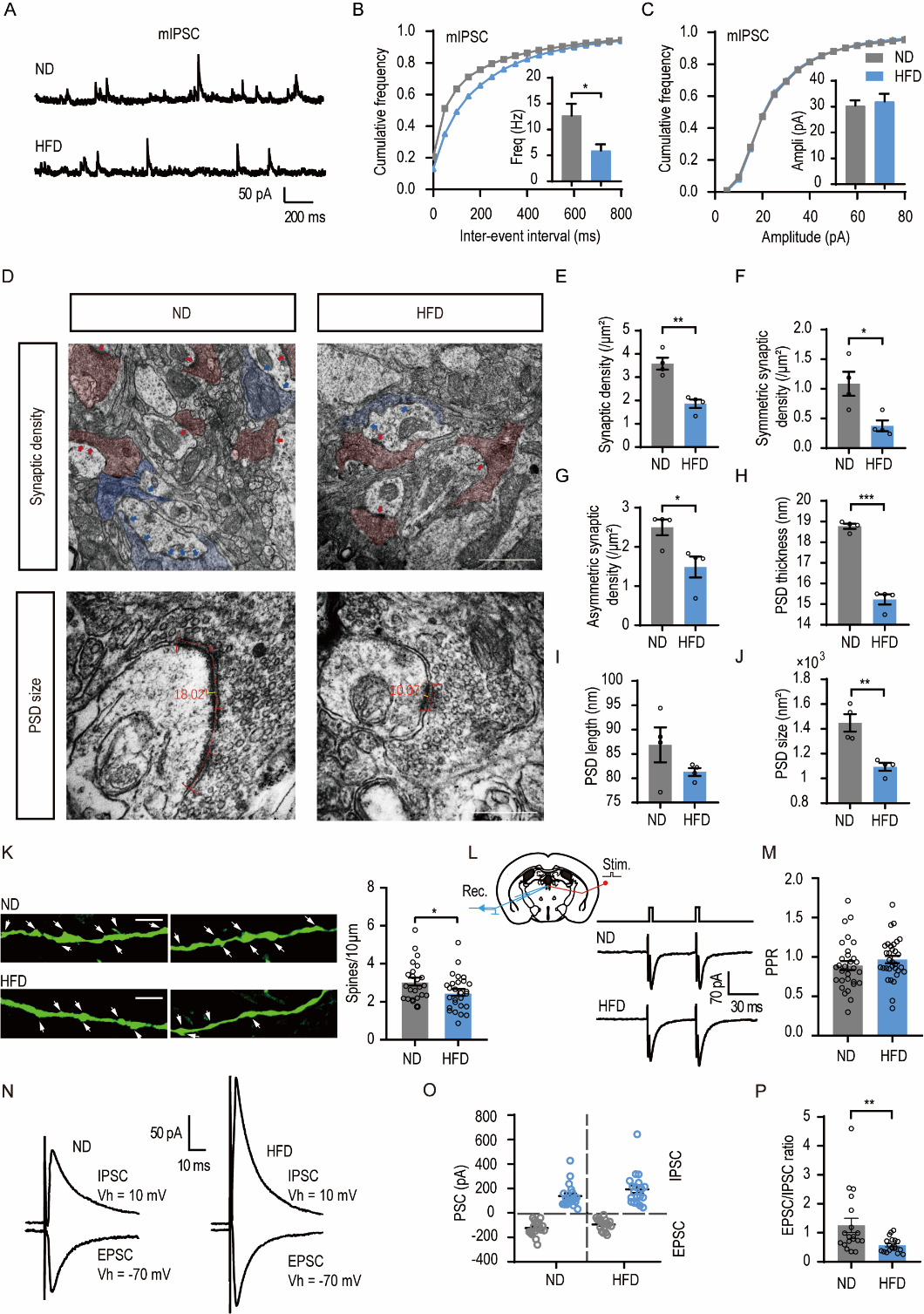


**Figure S5. AL HFD feeding decreases synaptic transmission** **and remodels E/I balance in PVT.**

(A) Representative mIPSC traces recorded from PVT neurons in ND and HFD mice in presence of TTX (1 µM).

(B) Cumulative fraction curves of inter-event intervals. Inset, mean frequency of mIPSCs (n = 4 mice, 17–19 cells, Mann-Whitney test).

(C) Cumulative fraction curves of amplitudes. Inset, mean amplitude of mIPSCs (n = 4 mice, 17–19 cells, Mann-Whitney test).

(D) Electron micrographs from PVT areas in ND (left) and HFD (right) mice. Top panel, Representative image of synaptic distributions. Different colored markers show different synaptic types, including symmetric synapse (blue) and asymmetric synapse (red). Scale bar, 300 nm. Bottom panel, Representative image of PSD. Scale bar, 100 nm.

(E-G) Mean of synaptic density (E), symmetric synaptic density (F), and asymmetric synaptic density (G) in PVT (n = 5 mice, two-tailed unpaired *t* test).

(H-J) Mean of PSD thickness (H), length (I), and size (J) of PVT synapses (n = 5 mice, two-tailed unpaired *t* test).

(K) Representative images of dendritic segments in the PVT of ND and HFD mice (left). Arrowheads indicate spines. Scale bar = 5 µm. Mean spine density of PVT neurons for ND and HFD mice (right, n = 8, two-tailed unpaired *t* test).

(L) Schematic of the experiment (left). Examples of EPSCs evoked by paired stimuli with inter-pulse interval of 50 ms in PVT of ND and HFD mice (right).

(M) Paired-pulse ratio (PPR, pulse 2/pulse 1) was not altered after chronic HFD (n = 5–6 mice, 30–32 cells, Mann-Whitney test).

(N) Representative traces of evoked IPSCs (top) and EPSCs (bottom) of PVT neurons in ND and HFD mice. Extracellular stimulation-evoked EPSCs and IPSCs monitored at −70 and 10 mV, respectively, in voltage-clamp mode.

(O) Mean amplitudes of evoked EPSCs and IPSCs in PVT neurons (n = 4 mice, 19 cells, two-tailed unpaired *t* test).

(P) Ratio between evoked EPSC and IPSC amplitudes in PVT neurons in ND and HFD mice (n = 4 mice, 19 cells, Mann-Whitney test).

Data are means ± SEM. For E to J, dots represent individual experimental animals. For K to P, dots represent individual experimental cells. **p* < 0.05; ***p* < 0.01; ****p* < 0.001 as indicated.

**Figure S6 related to Figure 2**


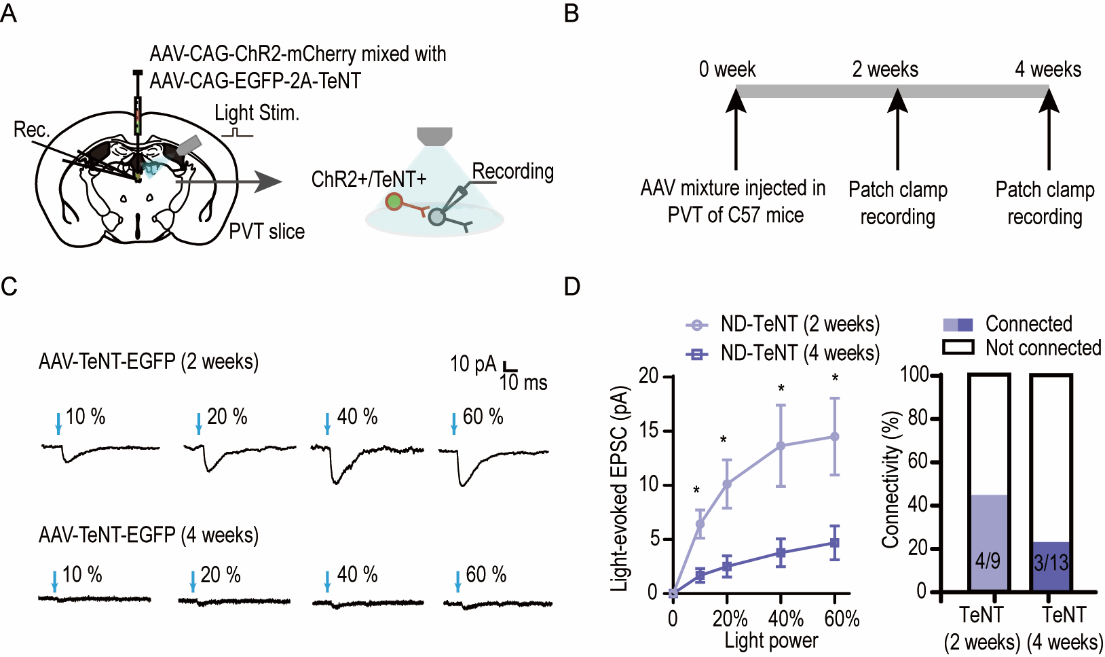


**Figure S6. Neurotransmitter release of PVT is blocked after expression of TeNT for 4 weeks.**

(A-B) Experimental design.

(C) Representative traces.

(D) Input-output curves of light-evoked EPSCs recorded from ChR2-negative neurons in PVT (left). Percentage of responding neurons to light (right).

Data are means ± SEM. For all panels, n = 3 mice, 9–13 cells, two-tailed unpaired *t* test was employed, **p* < 0.05.

**Figure S7 related to Figure 3**


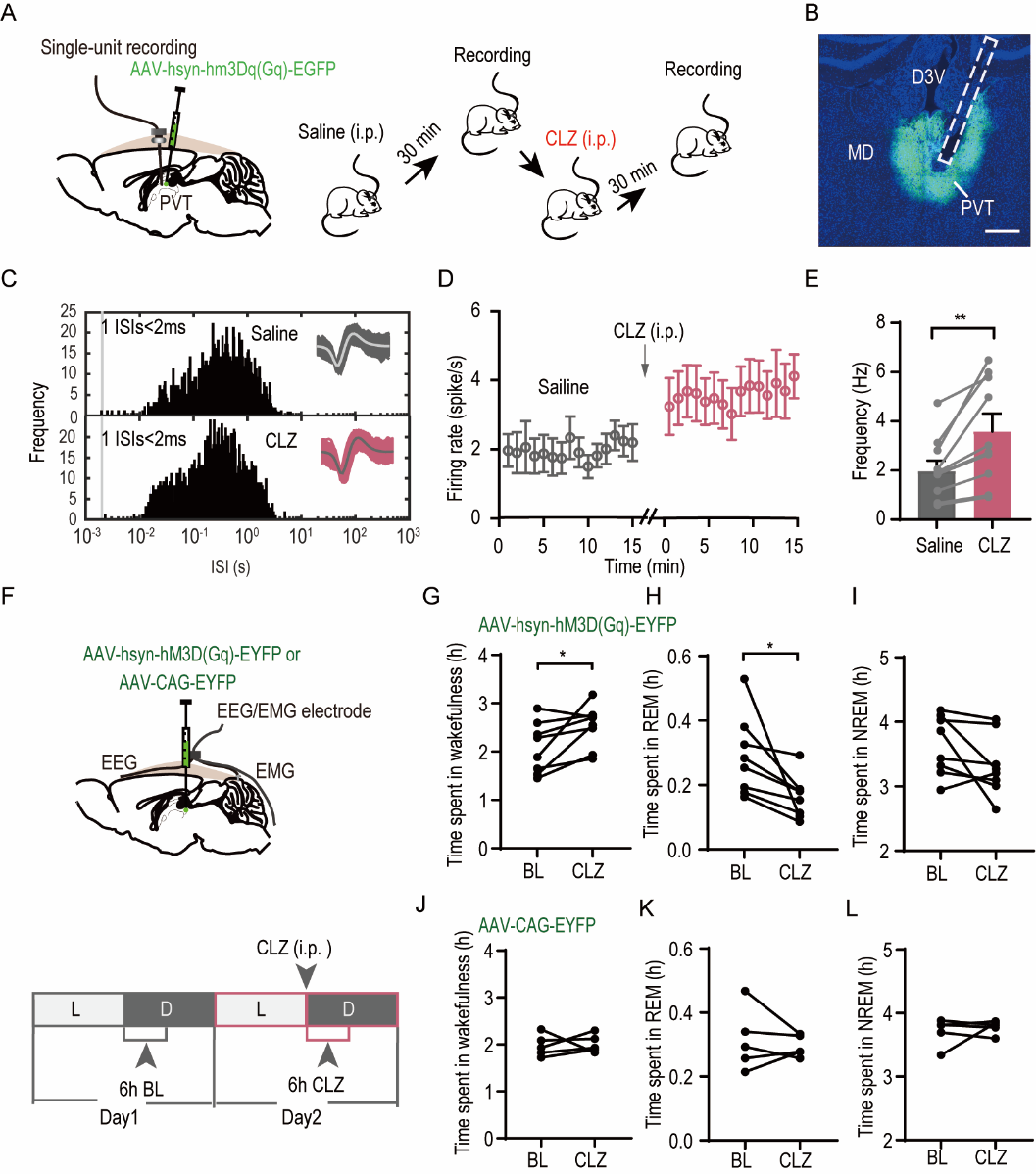


**Figure S7.** **Acute activation of PVT neurons increases wakefulness duration.**

(A) Schematic of virus injection and electrophysiological recording experiment.

(B) Representative image of electrode trace and hM3dq-EYFP expression. Scale bar, 300 μm.

(C) Histograms of inter-spike intervals (ISI) from same single unit under saline (top) and CLZ (bottom) conditions. Insets at top right corner show waveforms of single unit detected.

(D-E) CLZ administration increased PVT spike firing, as illustrated by change in time-averaged firing rate (D) and mean firing rate (E) (n = 10, two-tailed paired *t* test).

(F) Schematic of CLZ administration. EEG/EMG of baseline (BL) was recorded at night (6-hr, ZT12–ZT18), CLZ was administered at beginning of the night (ZT12) and the next day, then EEG/EMG was recorded for the next 6-hr (ZT12–ZT18).

(G-I) Total time spent in wakefulness (G), REM sleep (H), and NREM sleep (I) during 6-hr recording of AAV2-hsyn-CAG-hM3Dq-EYFP group (n = 8, two-tailed paired *t* test).

(J-L) Total time spent in wakefulness (J), REM sleep (K), and NREM sleep (L) during 6-hr recording of AAV-CAG-EYFP control group (n = 5, two-tailed paired *t* test).

Data are means ± SEM. For E, dots represent individual experimental cells. For G to L, dots represent individual experimental animals. **p* < 0.05; ***p* < 0.01 as indicated.

**Figure S8 related to Figure 4**


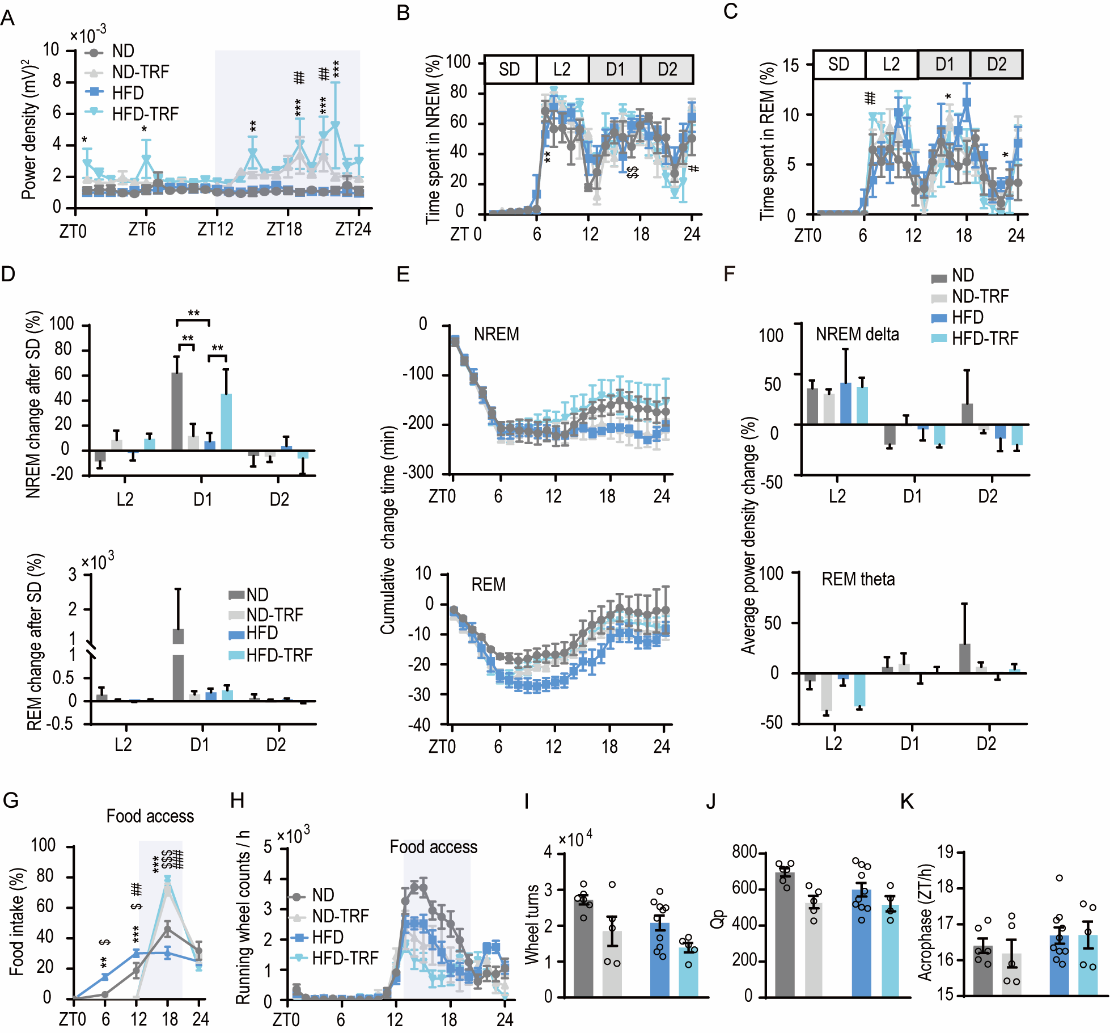


**Figure S8. Effects of TRF on circadian and homeostatic processes of sleep regulation.**

(A) Time course of EEG delta power (1.0–4.0 Hz) during NREM sleep across light (left) and dark (right) phases of ND, HFD, ND-TRF, and HFD-TRF mice (n = 9–12, two-way ANOVA, Bonferroni post hoc analysis).

(B, C) Percentage of time spent in NREM (B) and REM (C) sleep per hour during and after sleep deprivation (n = 4–5, two-way ANOVA, Bonferroni post hoc analysis).

(D) Percent change of sleep duration after sleep deprivation compared with the baseline for NREM (top) and REM (bottom) sleep in L2, D1, and D2 (n = 4–5, two-way ANOVA, Bonferroni post hoc analysis).

(E) Cumulative NREM (top) and REM (bottom) sleep loss and gain compared with baseline conditions for the sleep deprivation experiment.

(F) Analysis of NREM (top) and REM (bottom) sleep spectral power changes compared with baseline during L2, D1, and D2, respectively (n = 4–5, two-way ANOVA, Bonferroni post hoc analysis).

(G) Food ingested under light: dark cycle (LD; 12 hr:12 hr). (n = 5, two-way ANOVA, Bonferroni post hoc analysis).

(H) Number of wheel revolutions over 24-hr LD cycle when given access to a running wheel in home cage for the four groups (ND, ND-TRF, HFD, HFD-TRF).

(I) Average number of wheel revolutions over 24-hr LD cycle (n = 5–10, two-way ANOVA, Bonferroni post hoc analysis).

(J) Periodogram power measured by chi-squared periodogram analysis (n = 5–10, two-way ANOVA, Bonferroni post hoc analysis).

(K) Time at which the peak of wheel-running rhythm occurred measured by cosinor analysis (n = 5–10, two-way ANOVA, Bonferroni post hoc analysis).

Data are means ± SEM. Dots represent individual experimental animals. For B, C and G, significant differences between HFD and HFD-TRF are marked with “*”, differences between ND and ND-TRF are marked with “#”, differences between HFD and ND are marked with “$”. **p* < 0.05; ***p* < 0.01; ****p* < 0.001 as indicated.
