# Supplementary material for: Time-restricted feeding is an intervention against excessive dark-phase sleepiness induced by obesogenic diet
Source: Natl Sci Rev. 2022 Oct 16;10(1):nwac222. doi: 10.1093/nsr/nwac222 (PMC9942665; doi:10.1093/nsr/nwac222)
Supplement: nwac222_Supplemental_Files [file nwac222_supplemental_files.zip › Supplementary_Meterials_and_Methods.docx]

**METERALS AND METHODS**

## Animals

In our experiments, 6–7-week-old male C57BL/6J mice were allocated into two groups with AL access to either a ND (1032, Beijing HFK Bioscience, 20% protein, 4% fat, 68% carbohydrates) or a HFD (MD12033, Medicine, 20% protein, 60% fat, 20% carbohydrates) for 2 weeks to acclimatize to the diet. Then the mice were further subdivided into AL and TRF groups for each diet (In Fig. 6–7, AL or TRF were performed following 8 weeks of AL feeding). After 8 weeks of feeding treatments, mice were implanted with EEG-EMG, and injected with AAV. And then sleep measurements and neural activity manipulation experiments were conducted after recovering from surgeries. In addition, wheel-running activity, synaptic transmission, body weight, and food intake were all measured after 8 weeks of feeding treatments except for those noted otherwise. Mice were 16-25 weeks old by the end of the experiment.

Mice were kept in the Wuhan National Laboratory with regular temperature (22 ± 2°C), humidity (40%–60%) and circadian cycle (12:12 LD cycle, with light on at zeitgeber time 0 (ZT 0, 7:00 AM)). Water was provided AL. All experiments were performed in accordance with the guidelines of the Institute of Neuroscience, Chinese Academy of Sciences and University. All procedures involving animals were approved by the Hubei Provincial Animal Care and Use Committee and were in accordance with the experimental guidelines of the Animal Experimentation Ethics Committee of Huazhong University of Science and Technology, China.

## Stereotactic surgery

AAV-CAG-TeNT-2A-EYFP was provided by Man Jiang Lab (Department of Neurobiology, School of Basic Medicine and Tongji Medical College, Huazhong University of Science and Technology, Wuhan, China). Viruses were injected into PVT at the following stereotaxic coordinates: 0.85 mm from bregma, 0.8 mm lateral from midline, and 3.25 mm vertical from cortical surface with 15°angle. The experiments involved the following viruses: AAV-CAG-EGFP-2A-TeNT (AAV2/9, titer, 5.2$\times$10^13^ particles per ml); AAV-CAG-ChR2-mCherry (AAV2, titer, 3.9$\times$10^13^ particles per ml); AAV-hsyn-CAG-hM3Dq-EYFP (AAV2/9, titer, 4$\times$1011 particles per ml); AAV-CAG-EYFP (AAV9, 2$\times$10^12^ particles per ml).

## EEG-EMG electrode implantation surgery

EEG recording stainless steel screws were inserted over the skulls (1.75 mm from bregma, 0.4 mm from bregma and reference electrode: cerebellum) and EMG recording wires were inserted into neck muscles as described previously [1]. The EEG-EMG electrodes were affixed to the skulls using Super-Bond C&B and dental acrylic. The mice were allowed to recover for at least 7 days.

## EEG-EMG recordings and analysis

After recovered from EEG/EMG implantation surgery, mice were connected to flexible EEG-EMG connection cables via slip rings. Then the mice were adapted in regular recording cages for at least 3 days with a 12:12 LD cycle. Illumination intensity is 100 lux during our recording. Food and water were available AL. A Microelectrode AC Amplifier Model 1700 (A-M System, USA) was used for EEG/EMG data acquisition, filtered (0.1–500 Hz or 10–500 Hz for EEG and EMG recordings, respectively). EEG/EMG signals were digitized through the Chart software (Plexon, TX, USA) with a sampling rate of 250 Hz. Mice which lost EEG and EMG signals during experiments were excluded. Sleep-wake state classification was manually performed based on EEG/EMG signals with MATLAB. States were assigned consecutive 4 s epochs. Wakefulness was defined as desynchronized, low-amplitude, and high-frequency EEG rhythms with elevated EMG activity. NREM sleep was defined as synchronized, high-amplitude, and low-frequency (0.5–4 Hz) EEG activity with lower tonic EMG tone. REM sleep was defined as containing prominent theta (4–9 Hz) oscillations on EEG and minimal EMG activity. For the analysis of power spectral, NREM delta and REM theta EEG powers were analyzed with fast Fourier transform (FFT) [2].

## *in vivo* single-unit recording

In vivo single-unit recordings were performed and analyzed as previous studies [1]. All recordings were performed in freely behaving animals. The electrodes consist of sixteen 25.4 μm diameter formvar-insulated nichrome wires (cat no.761500, A-M System, USA), and the wires were cut with sharp scissors. The final impedance of the electrodes was 700–800 kΩ. 16-channel electrodes were implanted in PVT area, the majority of recordings were from cells in target area. Electrophysiological recordings were not initiated until 7 days after surgery. Before testing, mice were adapted to recording chambers at least 3 days. During 4-hr recording, the 16-channel electrodes were connected to the amplifier and sampled by a computer. All signals were amplified (200 000 gain) and with a sampling rate of 40 kHz by the NeuroPhys Acquisition System (Neurosys 2.8.0.8, USA) and NeuroLego System (Jiangsu Brain Medical Technology Co.ltd).

## *in vivo* single-unit recording data analysis

Single-unit spike sorting was performed using the MATLAB toolbox (MClust-4.4). Cells with waveform amplitudes smaller than three times noise band were excluded from the data. Waveform features and distribution of spike timing were analyzed to sort the single-unit signals. First, waveforms peak value and two types of principal components were analyzed. Similar characters of waveforms were defined into clusters. Then, single-unit was defined by isolation distance (> 20), L-ratio (< 0.1), and inter-spike-interval (ISI) under 2 ms < 1% [3]. Units with mean firing rates < 0.5 Hz were excluded from analyses.

Neuronal activity and EEG/EMG were recorded simultaneously. The neuronal activity was matched with wakefulness, NREM sleep, and REM sleep states. To quantify the relative firing rates of each neuron in different brain states, we then plotted REM-NREM modulation ((R_REM_-R_NREM_) / (R_REM_+ R_NREM_)) versus wake-NREM modulation ((R_wake_-R_NREM_) / (R_wake_+R_NREM_)), where R represents the mean firing rate in each state.

## Patch clamp recording

Mice were deeply anesthetized with isoflurane and their brains were rapidly dissected from the skulls. Then the brains were placed in the cutting buffer contained the following (in mM): 2.5 KCl, 0.5 CaCl_2_, 7.2 MgCl_2_, 25 NaHCO_3_, 1.1 NaH_2_PO_4_, 25 D-glucose, 11 sodium ascorbate, 3 sodium pyruvate, and 97 choline chloride. Coronal slices (300 μm) were cut in 95% O_2_ /5% CO_2_-oxygenated cutting buffer with a vibratome (Leica VT1000S, Germany). Slices were incubated at 32°C in a submerged chamber containing ACSF equilibrated with 95% O_2_/5% CO_2_ for at least 30 min and followed by recovery for 30 min at room temperature. The ACSF contained the following (in mM): 118 NaCl, 2.5 KCl, 2 CaCl_2_, 2 MgCl_2_, 26 NaHCO_3_, 0.9 NaH_2_PO_4_, and 11 D-glucose. The slices bubbled with 95% O_2_/5% CO_2_ were transferred to a recording chamber. Patch-clamp electrodes were pulled from borosilicate glass (1.5 mm diameter, VitalSense, B15024N) on a three-stage puller (Sutter P-1000). Pipettes had a resistance of 2–6 MΩ. Recordings were collected using Axon Multiclamp 700B amplifiers, Digidata 1440A (Molecular Devices), and analyzed using MiniAnalysis software (Synaptosoft), Clampfit 10.3 software (Molecular Devices). During recordings, series resistances were continuously monitored. Recordings with series resistance of > 25 MΩ were excluded from analysis, we did not compensate the series resistance, but cells which Rs changed by > 15% were discarded [4]. For mEPSC recordings, the pipette solution contained the following (in mM): 140 K-gluconate, 0.1 CaCl_2_, 2 MgCl_2_, 1 EGTA, 2 ATP K_2_, 0.1 GTP Na_3_, and 10 HEPES (pH = 7.25, osmolality 305 mOsm). The mEPSCs were recorded for 10 min at a holding potential of -70 mV. Picrotoxin (10 μM) was added to the external solution to block GABA_A_ receptors. For mIPSC recordings, the pipette solution contained the following (in mM): 127.5 caesium methanesulfonate, 7.5 CsCl, 10 HEPES, 2.5 MgCl_2_, 4 Na_2_ATP, 0.4 Na_3_GTP, 10 sodium phosphocreatine, 0.6 EGTA (pH = 7.25, osmolality 305 mOsm). The mIPSCs were recorded for 10 min at a holding potential of 10 mV. D-AP5 (20 μM, Tocris Bioscience) and CNQX (10 μM, Tocris Bioscience) were added to the external solution to block NMDARs and AMPARs. All experiments were performed in the presence of tetrodotoxin (TTX, 1 μM). To test the E/I ratio within the PVT, excitatory and inhibitory postsynaptic currents were recorded by holding the membrane potential under - 70 mV and 10 mV respectively. Postsynaptic currents were evoked through a bipolar stimulating electrode (20 ms, 1 Hz, 15 trails, MicroProbes) placed in PVT. PPR was measured by applying stimuli at an inter-pulse interval of 50 ms, with the stimulation electrode placed within the PVT. The stimulation intensity was kept constant over the whole recording duration. The intrinsic membrane properties were measured via current injections under current-clamp mode. Depolarizing current steps of 500 ms duration were applied in 3–10 pA increments. The following parameters were measured: RMP, spike threshold, first spike latency and rheobase current.

The AAV-mediated optogenetics approach was used to investigate the efficiency of inactivation of PVT with tetanus neurotoxin (TeNT) on neuronal activity. In this experiment, we injected a mixture of 200nl AAV-CAG-EGFP-2A-TeNT and 200nl AAV-CAG-ChR2-mCherry into the PVT, resulting in the co-expression of TeNT-EGFP and ChR2-mCherry in the same neuron. Patch-clamp recordings were performed after virus expression for 2 and 4 weeks. During the recordings, light (473 nm, 0.5 ms) evoked action potentials in ChR2-mCherry-positive cells, and postsynaptic currents were recorded from ChR2-mCherry-negative PVT neurons triggered by single light pulses. This method is similar to that in previous publication [5].

## Electron microscopy

The procedure was according to previous works [6, 7]. Mice were deeply anesthetized with sodium pentobarbital, and then 50 mL of 0.01 M phosphate buffered saline (PBS; pH 7.4), 100 mL of 4% (w/v) paraformaldehyde and 0.05% glutaraldehyde in 0.1 M PB (pH 7.4) were used for transcardial perfusion. After perfusion, the brains were placed in 3% glutaraldehyde for an additional 2-h. 50 μm sections containing PVT were then serially cut with a vibratome. And then, the sections were treated with 1% OsO_4_ in 0.1 M PB for 45 min. The sections containing PVT were flat-embedded in epoxy resin after dehydration. After the resin polymerized, PVT was identified and excised from the sections under a light microscope. The selected tissue was further cut into 70 nm ultrathin sections with a diamond knife mounted on an ultramicrotome. The ultrathin sections were stained with 1% (w/v) lead citrate, and then examined with an electron microscope. Synapses with typical presynaptic structures (with vesicles) and postsynaptic structures (with PSDs) were collected. In addition, the PSD thickness and length were measured.

## Imaging and analysis of spines

AAV-CAG-EYFP (AAV9, 2$\times$10^12^ particles per ml) was injected into the PVT, and spines were analyzed after EYFP expression. Serial coronal sections were prepared using a Leica VT1200S vibratome. Intact dendritic branches (at least 20 µm, 1-3 branches per neuron) parallel to the section plane were scanned with the Nikon AXR confocal microscope and the 100$\times$ oil-immersion objective with a z-step size of 0.2 µm.

## Chemogenetic manipulations

AAV-hysn-hM3D (Gq)-EYFP were injected in PVT for HFD fed mice. After 3 weeks recovery, EEG-EMG electrodes were implanted over the brain. The virus expression for four weeks, EEG and EMG signals were recorded for hM3Dq-EYFP or EYFP expression mice. Clozapine (CLZ, selective ligand for hM3Dq, 0.1 mg kg^-1^ dissolved in saline) was intraperitoneally injected at ZT13 (the starting of lights off) for hM3Dq-EYFP or EYFP expression mice. After 2 weeks of CLZ injection, EEG and EMG signals were recorded again.

## Food intake assay

Mice were first single housed for at least 3 days with a 12:12 LD cycle. Food and water were available AL. And then food intakes were monitored every 6-h during light phase, starting at lights on (ZT0).

## Wheel running

Mice were singly housed in mice’s home cages, equipped with a 12 cm diameter running wheel (magnetic switch is installed). Then, mice were adapted in the running wheel under a 12:12 h LD cycle for at least 7 days, followed by recording for 3 days, with ad libitum access to food and water. The number of activity bouts per day was counted by a behavioral operating system (LabState ver1.0, AniLab Software & Instruments, China). The amplitudes of daily activity rhythms were calculated using a cosinor analysis [8] (<https://cosinor.online/app/cosinor.php>). To estimate the robustness of the wheel-running rhythms, the periodogram curve of 6 days of wheel-running activties were measured using the chi-square periodogram (ImageJ), and the periodogram power is the value at the top of the curve (*Q*p). The animals used in the wheel-running test were not used in other experiments.

## Sleep deprivation

Mice were allowed to adapt in regular recording chambers for at least 3 days. Then, baseline (BL) days were recorded, starting at lights on (ZT0). At the start of the second day, 6-hr of sleep deprivation was conducted by gentle handling. If an animal appeared drowsy or the EEG exhibited slow waves for a few seconds. We gently agitated the animal with a plastic rod to wake it up [9]. EEG and EMG were recorded continuously during sleep deprivation and the 18-hr after sleep deprivation.

## Glucose tolerance

Mice were fasted for 16-h, and fasted blood glucose was measured using a Glucometer (Accu-Chek Active) by tail bleeds. Mice were intraperitoneally injected with glucose (1 g/kg of body weight in saline), blood glucose was continuously monitored for 2-h.

## QUANTIFICATION AND STATISTICAL ANALYSIS

Statistical tests included Mann-Whitney test, Kolmogorov-Smirnov test, Chi-square test, two-tailed unpaired *t* tests, two-way paired *t* tests, and two-way ANOVA. Analyses were conducted using the GraphPad Prism 8 (GraphPad Software) statistical software for Windows, SPSS software (v.22, IBM, New York, NY, USA), or MATLAB R2014b (Mathworks). Data are expressed as mean ± SEM. Statistical significance was set at **p* < 0.05; ***p* < 0.01; ****p* < 0.001.

## REFERENCES

[1] R. F. Hua, X. Wang, X. F. Chen, X. X. Wang, P. C. Huang, P. C. Li, et al. Calretinin Neurons in the Midline Thalamus Modulate Starvation-Induced Arousal. Current Biology, 2018, 28(24): 3948-3959

[2] G. S. Shi, L. J. Xing, D. Wu, B. J. Bhattacharyya, C. R. Jones, T. McMahon, et al. A Rare Mutation of beta(1)-Adrenergic Receptor Affects Sleep/Wake Behaviors. Neuron, 2019, 103(6): 1044-+

[3] P. Huang, X. Xiang, X. Chen and H. Li. Somatostatin Neurons Govern Theta Oscillations Induced by Salient Visual Signals. Cell Rep, 2020, 33(8): 108415

[4] X. X. Wang, Y. S. Zhang, X. Wang, J. Q. Dai, R. F. Hua, S. Q. Zeng, et al. Anxiety-related cell-type-specific neural circuits in the anterior-dorsal bed nucleus of the stria terminalis. Science Bulletin, 2020, 65(14): 1203-1216

[5] C. Shang, Z. Chen, A. Liu, Y. Li, J. Zhang, B. Qu, et al. Divergent midbrain circuits orchestrate escape and freezing responses to looming stimuli in mice. Nat Commun, 2018, 9(1): 1232

[6] J. J. Wang, X. Y. Lv, Y. Wu, T. Xu, M. F. Jiao, R. S. Yang, et al. Postsynaptic RIM1 modulates synaptic function by facilitating membrane delivery of recycling NMDARs in hippocampal neurons. Nature Communications, 2018, 9

[7] T. Chen, J. Li, B. Feng, R. Hui, Y. L. Dong, F. Q. Huo, et al. Mechanism Underlying the Analgesic Effect Exerted by Endomorphin-1 in the rat Ventrolateral Periaqueductal Gray. Mol Neurobiol, 2016, 53(3): 2036-2053

[8] W. Nelson, Y. L. Tong, J. K. Lee and F. Halberg. Methods for cosinor-rhythmometry. Chronobiologia, 1979, 6(4): 305-23

[9] T. Deboer, H. C. van Diepen, M. D. Ferrari, A. M. J. M. Van den Maagdenberg and J. H. Meijer. Reduced Sleep and Low Adenosinergic Sensitivity in Cacna1a R192Q Mutant Mice. Sleep, 2013, 36(1): 127-136
